# Metabolic stress sensing by epithelial RXRα links westernization of diet with Crohn’s disease

**DOI:** 10.1016/j.cmet.2025.11.008

**Published:** 2025-12-08

**Authors:** Moritz Meyer, Felix Grabherr, Christina Plattner, Michel V. Hadjihannas, Zhigang Rao, Valentin Marteau, Víctor Alonso López-Agudelo, Julian Schwärzler, Lisa Mayr, Almina Jukic, Laura Scheffauer, Luis Zundel, Barbara Enrich, Alexandra Pfister, Anna Simonini, Christoph Grander, Richard Hilbe, David Haschka, Andreas Zollner, Kathrin Vouk, Patrizia Moser, Michael W. Hess, Niloofar Nemati, Dietmar Rieder, Felix Sommer, Philip Rosenstiel, Qitao Ran, Richard S. Blumberg, Arthur Kaser, Florian Rieder, Andreas Koeberle, Christoph Becker, Raja Atreya, Anja A. Kühl, Britta Siegmund, Andre Franke, The IBDome Consortium, Herbert Tilg, Zlatko Trajanoski, Timon E. Adolph

**Affiliations:** 1 Department of Internal Medicine I, Gastroenterology, Hepatology, Endocrinology & Metabolism, Medical University of Innsbruck, Innsbruck, Austria; 2 Biocenter, Institute of Bioinformatics, Medical University of Innsbruck, Innsbruck, Austria; 3 Institute of Clinical Molecular Biology, Christian Albrechts University Kiel and Schleswig-Holstein University Hospital, Kiel, Germany; 4 Michael Popp Institute and Center for Molecular Biosciences Innsbruck (CMBI), University of Innsbruck, Innsbruck, Austria; 5 Department of Internal Medicine II, Infectious Diseases, Immunology, Rheumatology, Pneumology, Medical University of Innsbruck, Innsbruck, Austria; 6 INNPATH, Innsbruck Medical University Hospital, Innsbruck, Austria; 7 Institute of Histology and Embryology, Medical University of Innsbruck, Innsbruck, Austria; 8 Department of Cell Systems and Anatomy, UT Health San Antonio, San Antonio, TX, USA; 9 Gastroenterology Division, Department of Medicine, Brigham and Women’s Hospital, Harvard Medical School, Boston, MA, USA; 10 Division of Gastroenterology and Hepatology, Department of Medicine, Addenbrooke’s Hospital, University of Cambridge, Cambridge, UK; 11 Department of Gastroenterology, Hepatology & Nutrition, Digestive Diseases Institute, Department of Inflammation and Immunity, Lerner Research Institute, Center for Global Translational Inflammatory Bowel Disease Research, Cleveland Clinic Foundation, Cleveland, OH, USA; 12 Institute of Pharmaceutical Sciences and Excellence Field BioHealth, NAWI Graz, University of Graz, Graz, Austria; 13 Department of Medicine 1, Friedrich-Alexander University Erlangen-Nürnberg, Erlangen, Germany; 14 Charité – Universitätsmedizin Berlin, Freie Universität Berlin and Humboldt-Universität zu Berlin, iPATH.Berlin, Berlin, Germany; 15 Charité – Universitätsmedizin Berlin, Freie Universität Berlin and Humboldt-Universität zu Berlin, Department of Gastroenterology, Infectious Diseases and Rheumatology, Berlin, Germany; 16 These authors contributed equally; 17 These authors contributed equally; 18 Lead contact

## Abstract

Westernization of diet, partly characterized by long-chain fatty acid excess, perturbs intestinal immune responses in Crohn’s disease (CD). The cellular and molecular framework of lipid sensing in intestinal inflammation remains enigmatic. By small intestinal transcriptional profiling of CD, we identified increased transcriptional activity of retinoid X receptor alpha (RXR**α**) specifically in intestinal epithelial cells (IECs). Transcriptional RXR**α** activity was induced in IECs of mice by **ω**−3 and **ω**−6 polyunsaturated fatty acid (PUFA) excess in a Western diet. PUFA-induced RXR**α** activity in Paneth cells governed chronic transmural enteritis by enabling the expression of CXCL1. Oral exposure to isotretinoin ameliorated PUFA-induced metabolic enteritis in two mouse models, and isotretinoin therapy reduced the odds of developing CD in an analysis of electronic health care records from 170,597 patients. Collectively, we identify RXR**α** in Paneth cells as a metabolic stress sensor that enables enteritis, providing novel perspectives for the prevention and treatment of CD.

## In brief

The long-chain fatty acid receptor RXR**α** exhibits transcriptional activation in the intestinal epithelium of patients with Crohn’s disease (CD). Dietary polyunsaturated fatty acids induce intestinal epithelial RXR**α** activity in susceptible mice, which instigates chronic enteritis emanating from Paneth cells. This study reveals a cellular and molecular framework for fatty acid sensing in intestinal inflammation, which could be exploited to prevent or treat CD.

## Graphical abstract

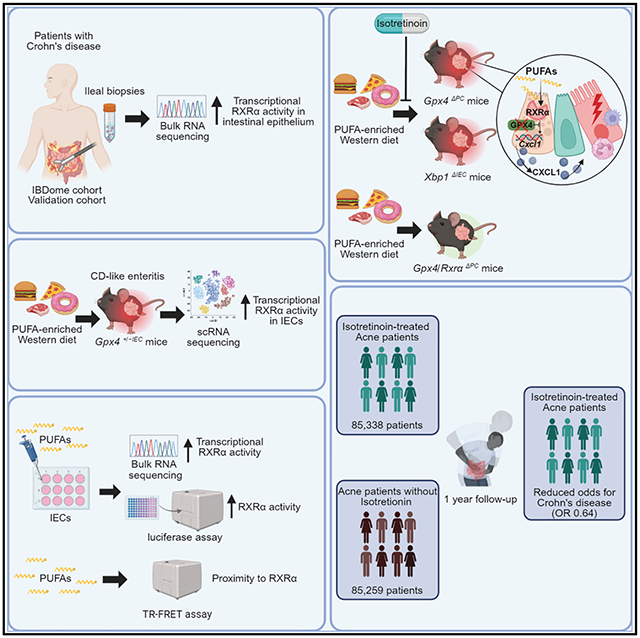

## INTRODUCTION

Crohn’s disease (CD) is a chronic remitting inflammatory condition affecting the gastrointestinal tract in more than 7 million people worldwide, and it is suspected that immunometabolism acts as an important disease mechanism in patients with CD.^1–3^ Immunometabolism of innate and adaptive immune cells describes a concept in which metabolic processes and cellular redox homeostasis shape mucosal immune responses, which can be influenced by diet and specific nutrients.^2,4,5^ Inflammatory bowel diseases (IBDs) and specifically CD rise in incidence globally during westernization of diet, which promotes perturbation of the intestinal microbiota,^6–8^ and emerges in an increasingly complex genetic landscape.^9,10^ Westernization of diet is characterized by a complex industrialized food pattern (as compared with the hunter-gatherer diet) and is partly characterized by an excess of long-chain fatty acids such as saturated and ω-6 polyunsaturated fatty acids (PUFAs),^11,12^ while ω-3 PUFAs are commonly supplemented by humans and in animal feed in Western countries.^13^ Consumption of meat and fat, and specifically ω-6 and ω-3 PUFAs, poses a risk for developing CD, but not ulcerative colitis, particularly in Asian populations.^14^ Moreover, saturated fat intake correlated with transcriptional markers of inflammation in the ileum of CD patients (e.g., expression of *S100A8*, *S100A9*, *TLR2/8*, and *CARD9*).^8^ As such, recent observations imply that the intestine is able to sense specific lipids that are enriched in a Western diet, which may lead to the orchestration of local inflammatory processes. A specific cellular and molecular framework for this concept, however, remains elusive.^2,15^

Paneth cells, specialized intestinal epithelial cells (IECs) with leukocyte-like functions,^16^ are originators of small intestinal inflammation in mice,^17^ and they have been implicated in the development and course of human CD.^18,19^ Paneth cells appear susceptible to environmental cues and specifically to a high-fat Western diet,^20,21^ but it is unknown if and how they sense long-chain fatty acids and whether this is controlling gut inflammation. Long-chain fatty acid sensing by mammalian cells is enabled by a variety of transcription factors, such as peroxisome proliferator-activated receptors and the retinoid X receptor alpha (RXRα).^22^ RXRα is a nuclear receptor for the PUFA docosahexaenoic acid (DHA) and serves as a transcription factor that controls gene expression involved in metabolism and immune responses.^23,24^ More specifically, RXRα has been implicated in the control of lipid metabolism, which is partly mediated by interactions with other nuclear receptors such as the retinoic acid receptor.^25^ Upon ligation with the PUFA DHA, RXRα homo- or heterodimerizes and induces gene transcription in the brain.^26–28^ RXRα is also involved in cholesterol metabolism,^29^ viral defense,^30^ and the production of macrophage chemoattractants.^24^ More recently, RXRα emerged as a transcription factor controlling differentiation of Paneth cells in the small intestine, and RXRα is involved in radiation-induced injury in the colon of mice (which is devoid of Paneth cells), which is controlled by retinol.^31^ A role for intestinal epithelial RXRα in dietary long-chain fatty acid sensing is not established but could contribute to IBDs.

By using bulk RNA sequencing of the small intestinal mucosa from 120 patients with CD and validation in independent cohorts at single-cell resolution,^32,33^ we report increased transcription factor activity of RXRα in the intestinal epithelium when compared with controls without IBD. In mice, transcriptional RXRα activity is induced in intestinal epithelium by PUFA excess in a Western diet, and dietary PUFAs elicit RXRα activity in intestinal epithelium. PUFA-induced RXRα activity governs chronic enteritis and CXCL1 production in Paneth cells. This molecular framework is druggable by oral isotretinoin treatment in mice and could be used to prevent or treat CD, as supported by the observation that oral isotretinoin treatment for dermatological indications^34^ reduces the odds of developing CD.

## RESULTS

### Small intestinal CD exhibits increased transcriptional RXRα activity in the intestinal epithelium

**Figure 1.**
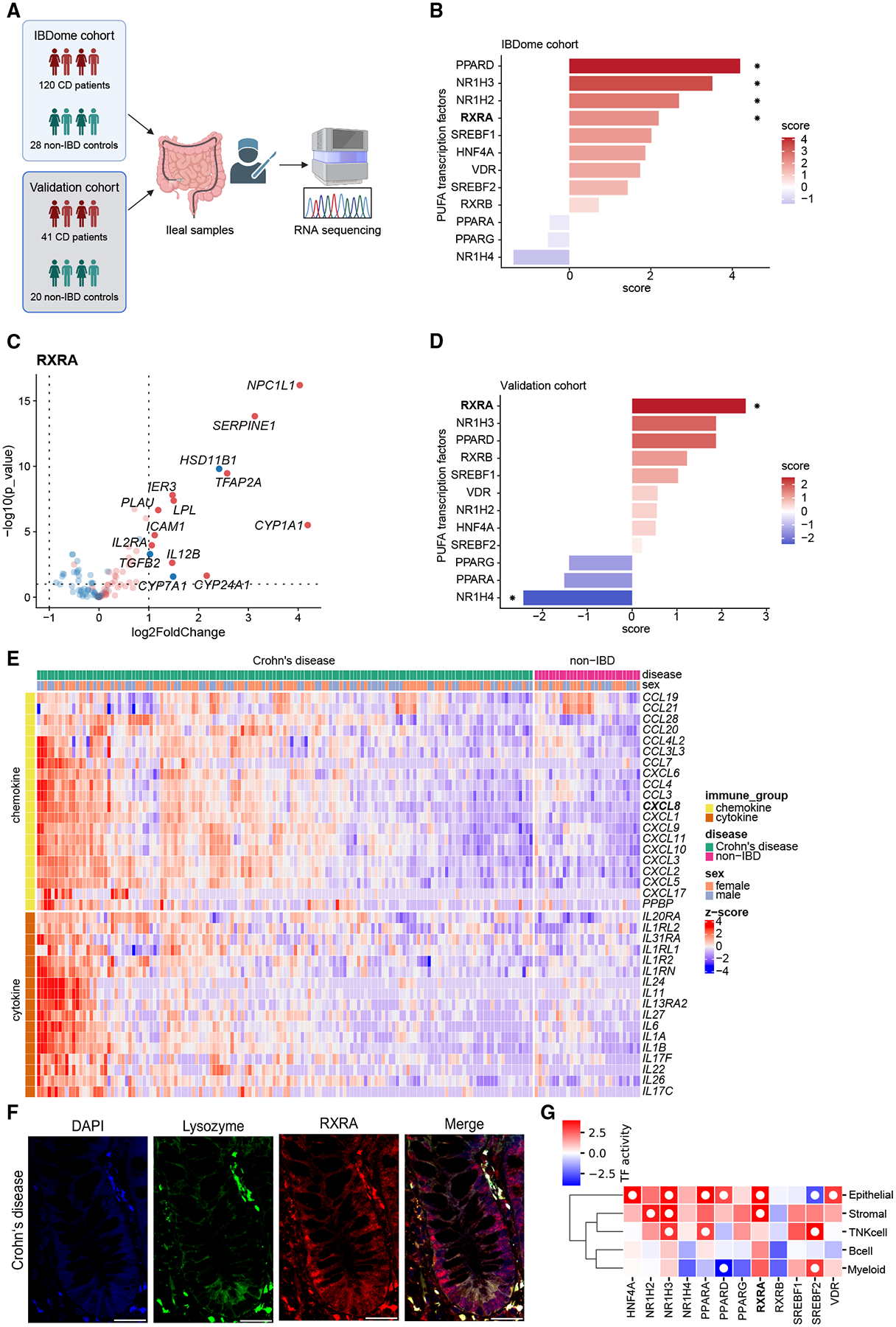
Transcriptional RXRα activity is induced in small intestinal CD epithelium (A) Illustration of the experimental approach. Bulk RNA sequencing of ileal CD specimens derived by colonoscopy or surgical resection was analyzed from the IBDome discovery cohort and a validation cohort.^32^ The IBDome cohort comprises 141 samples from 120 patients with CD and 30 non-IBD control samples from 28 individuals. The validation cohort comprises 46 intestinal samples from 41 patients with CD and 21 samples from 20 non-IBD controls. In both cohorts, transcription factor activity was inferred by their transcriptional profile. (B) Estimated transcription factor activities of long-chain fatty acid receptors in the IBDome cohort as assessed by a univariate linear model using CollecTRI in patients with small intestinal CD relative to non-IBD controls. (C) Volcano plot (color indicates the mode of regulation: inhibition, blue; activation, red) depicting RXRα target gene expression in the small intestinal mucosa of patients with CD compared with non-IBD controls in the IBDome cohort. (D) Estimated transcription factor activities of long-chain fatty acid receptors as assessed by a univariate linear model using CollecTRI in the validation cohort^32^ in patients with small intestinal CD relative to non-IBD controls. (E) Heatmap showing differentially expressed cytokines and chemokines in the small intestinal mucosa of patients with CD compared with non-IBD controls of the IBDome discovery cohort. (F) Representative confocal image of crypt epithelium in the small intestine from patients with CD (*n* = 3), with lysozyme (green) and RXRA (red) immunolabeling. Note the co-labeling of RXRα with lysozyme, indicating that Paneth cells express RXRα. DAPI indicates nuclei (blue). Scale bar, 50 μm. (G) Estimated transcription factor activities of long-chain fatty acid receptors in the different intestinal cell types from 13 patients with CD compared with 7 nonIBD controls as assessed by applying a univariate linear model using CollecTRI. Red color indicates upregulation of indicated transcription factor regulons relative to non-IBD controls. The white dot indicates statistical significance (FDR < 0.1). *FDR < 0.1.

We studied transcription factor activity of known long-chain fatty acid receptors in the IBDome discovery cohort, which comprises 141 ileum samples from 120 patients with CD and 30 ileum samples from 28 individuals without IBD (Figure 1A), with patient characteristics provided in Table S1. We inferred transcription factor activities with a univariate linear model using CollecTRI, a collection of validated transcriptional regulatory interactions.^35^ Our analysis revealed an ~2-fold increased transcription factor activity of RXRα in the small intestinal mucosa of patients with CD compared with non-IBD controls (Figure 1B). Specifically, 14 of 106 RXRα target genes exhibited significantly increased expression in the ileum of patients with CD (Figure 1C; Table S2). Transcriptional evidence for increased RXRα activity in small intestinal CD was validated in an independent cohort comprising 41 patients with CD and 20 non-IBD controls (Figures 1D and S1A).^32^ By contrast, increased activities of PPARD, NR1H3, and NR1H2 observed in the discovery cohort (Figure 1B) were not confirmed in the validation cohort (Figure 1D). Notably, in overweight/obese patients (indicated by a BMI ≥ 25), transcriptional RXRα activity was significantly increased as compared with patients with a BMI < 25 (Figure S1B). Increased transcriptional RXRα activity in the discovery cohort was furthermore associated with a transcriptional inflammatory profile of cytokines and chemokines, for example, interleukin 8 (IL-8, encoded by *CXCL8*) and activity of CD-related transcription factors (Figures 1E, S1C, and S1D), as similarly observed in the validation cohort (Figures S1E and S1F). The expression of *RXRA* was comparable between patients with CD and non-IBD controls in both cohorts (Figure S1G), and confocal imaging confirmed the expression of RXRα in gut epithelium and Paneth cells of patients with CD *in situ* (Figure 1F). In a next step, we analyzed a published single-cell sequencing dataset comprising 52,904 cells from inflamed tissue of patients with CD and 223,755 cells in total (with bowel segments of non-IBD patients serving as controls)^33^ and noted an increased transcriptional RXRα activity, but not RXRβ activity, in epithelium derived from the inflamed mucosa of patients with CD, as compared with non-IBD controls (Figure 1G). Collectively, these studies provide transcriptional evidence for increased RXRα activity in the mucosa of patients with CD, and specifically in intestinal epithelium.

### PUFA excess in a Western diet induces transcriptional RXRα activity in IECs of mice

**Figure 2.**
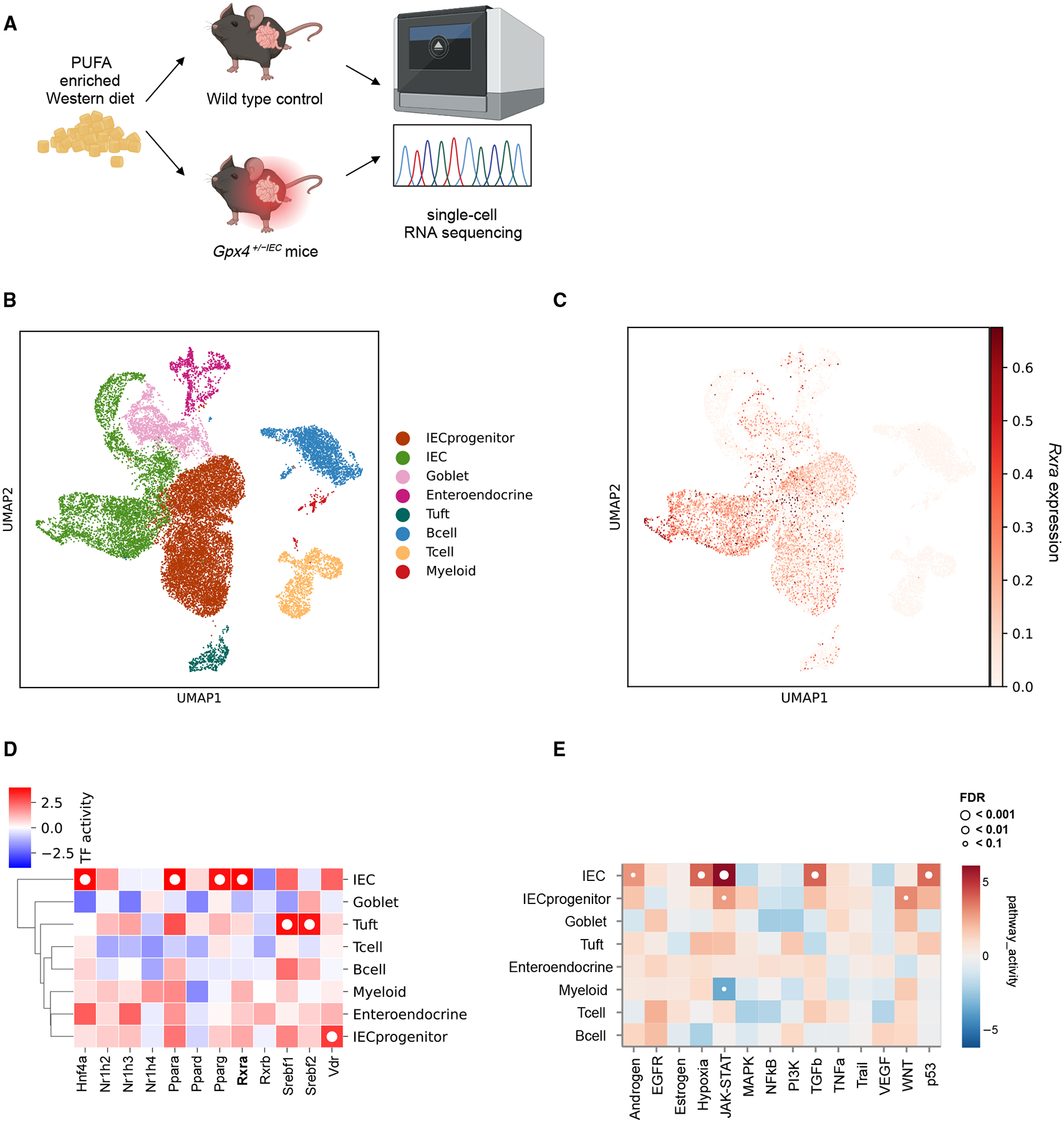
PUFA excess in a Western diet induces transcriptional RXRα activity in mouse IECs (A) Illustration of experimental design. *Gpx4*^+/−*IEC*^ mice and WT controls were fed a PUFA-enriched Western diet for 3 months. Single cells were extracted from small intestinal tissue and analyzed by single-cell RNA sequencing. (B and C) UMAP projection depicting different cell populations (B) and *Rxra* expression (C) of small intestinal samples from *Gpx4*^+/−*IEC*^ mice and WT controls (WT, *n* = 3; *Gpx4*^+/−*IEC*^, *n* = 4). (D) Heatmap showing differential transcription factor activity inferred by transcription factor regulons in mucosal cell populations from *Gpx4*^+/−*IEC*^ mice compared with WT controls (WT, *n* = 3; *Gpx4*^+/−*IEC*^, *n* = 4), computed using CollecTRI. Red color indicates upregulation of a transcription factor regulon relative to WT controls. The white dot indicates statistical significance (FDR < 0.1). (E) Heatmap showing differential pathway activity in mucosal cell populations from *Gpx4*^+/−*IEC*^ mice compared with WT controls (WT, *n* = 3, *Gpx4*^+/−*IEC*^, *n* = 4), computed using PROGENy. Red color indicates an increased pathway activity relative to WT controls. The white dot indicates statistical significance.

As RXRα may serve as a receptor for PUFAs,^23,27^ we next studied the origin of increased transcriptional RXRα activity in CD by using a model of Crohn’s-like enteritis that is induced by dietary PUFA excess.^36,37^ More specifically, we took advantage of a transgenic mouse that developed aspects of small intestinal CD after exposure to PUFA excess in a Western diet, but not a Western diet without PUFA enrichment, which was enabled by reduced (but not completely abrogated) intestinal epithelial activity of glutathione peroxidase 4 (GPX4) in *Gpx4*^+/−*IEC*^ mice.^36,37^ We assessed the transcriptional evidence for RXRα activity in the inflamed mucosa from *Gpx4*^+/−*IEC*^ mice by single-cell RNA sequencing after exposure to a PUFA-enriched Western diet for 3 months (Figure 2A) as compared with wild-type (WT) littermates without enteritis. Briefly, we analyzed 21,379 cells from *Gpx4*^+/−*IEC*^ and WT mice (Figure 2B) and defined IEC types as shown in Figure S2A. *Rxra* expression was confined to gut epithelial cells in *Gpx4*^+/−*IEC*^ mice (Figures 2C, S2B, and S2C). More importantly, we observed increased transcriptional RXRα activity in IECs from *Gpx4*^+/−*IEC*^ mice when compared with WT, as determined by CollecTRI analysis (Figure 2D), which was confirmed by qPCR of known RXRα-targeted genes in small intestinal scrapings of *Gpx4*^+/−*IEC*^ mice (Figures S2D–S2I). Transcriptional RXRα activity in IECs from *Gpx4*^+/−*IEC*^ mice was associated with activation of CD-related inflammatory pathways, for example, the JAK-STAT pathway (Figure 2E). These studies indicated that PUFA excess in a Western diet induced intestinal epithelial RXRα activity, which was restricted by GPX4 and associated with an inflammatory response in mammals.

### Dietary ω-3 and ω-6 PUFAs elicit intestinal epithelial RXRα activity

**Figure 3.**
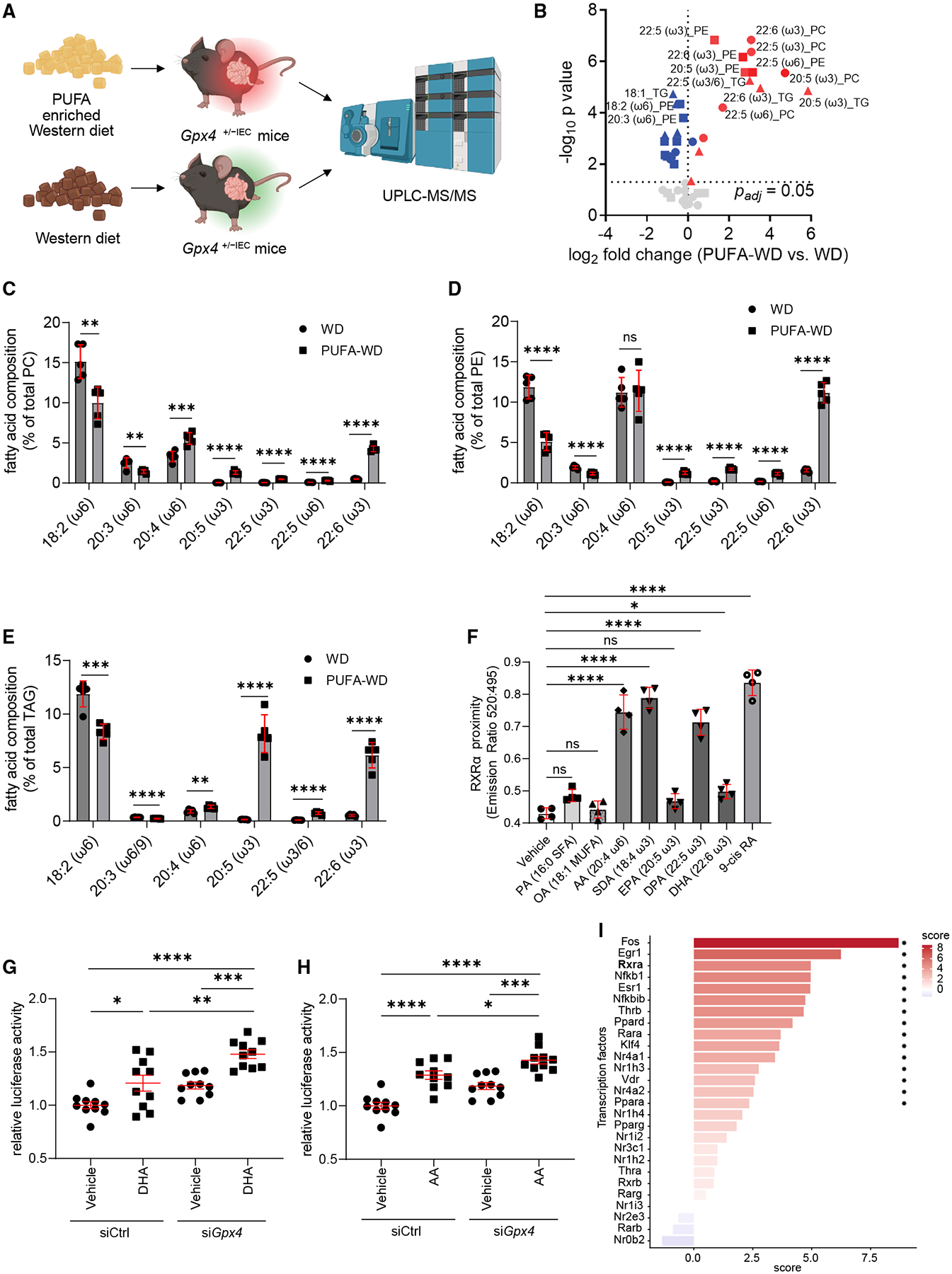
Dietary PUFAs elicit intestinal epithelial RXRα activity (A) Illustration of experimental design. *Gpx4*^+/−*IEC*^ mice were fed a PUFA-enriched Western diet or a Western diet without PUFA enrichment for 3 months to investigate by UPLC-MS/MS which lipid species are enriched in epithelial scrapings by the PUFA-enriched Western diet. (B) Volcano plot comparing differences in the proportions of the indicated fatty acids within PC, PE, or TG in small intestinal scrapings at the site of inflammation from *Gpx4*^+/−*IEC*^ mice fed a PUFA-enriched Western diet or a Western diet without PUFA enrichment for 3 months (*n* = 5). Red color depicts significantly up-regulated lipid species in mice exposed to a PUFA-enriched Western diet as compared with a Western diet. (C–E) Proportion of the indicated PUFAs within PC (C), PE (D), or TG (E) in the intestinal scrapings described under (B) (*n* = 5). (F) TR-FRET assay indicating close proximity of various lipids contained in the PUFA-enriched Western diet at a concentration of 1.85 μM with RXRα, as compared with vehicle or 9-*cis* retinoic acid at 1.85 μM after 3 h (*n* = 4). (G) Relative RXRα transcription factor activity, as assessed by a lentiviral luciferase reporter, in si*Gpx4* and siCtrl MODE-K IECs after stimulation with vehicle or docosahexaenoic acid (DHA) for 24 h (*n* = 10). (H) Relative RXRα transcription factor activity, as assessed by a lentiviral luciferase reporter assay, in si*Gpx4* and siCtrl IECs after stimulation with vehicle or arachidonic acid (AA) for 24 h (*n* = 10). Note that the vehicle group is the same as in (G). (I) Estimated transcription factor activity of AA-stimulated si*Gpx4* MODE-K IECs for 8 h, compared with siCtrl IECs as assessed by applying a univariate linear model using CollecTRI after bulk RNA sequencing (*n* = 6; 3 si*Gpx4* versus 3 siCtrl, Benjamini-Hochberg *p adjusted < 0.1). Data represented as mean (two-tailed multiple unpaired *t* tests with correction for multiple comparisons; false discovery rate [FDR] 1%) (B), mean ± SEM (two-tailed multiple unpaired *t* tests with correction for multiple comparisons; FDR 1%) (C–E), mean ± SEM (one-way ANOVA with post hoc Dunnett) (F), and mean ± SEM (one-way ANOVA with post hoc Bonferroni) (G and H). ns, not significant; *p < 0.05 or FDR < 0.1, **p < 0.01, ***p < 0.001, ****p < 0.0001.

Next, we investigated which PUFAs contained in the PUFA-enriched Western diet accumulated in epithelial fractions of mice and which of these directly elicited intestinal epithelial RXRα activity. To address this, we exposed *Gpx4*^+/−*IEC*^ mice to a PUFA-enriched Western diet or a Western diet without PUFA enrichment for 3 months. Phosphatidylcholines (PCs) and phosphatidylethanolamines (PEs), which are major phospholipids of cellular membranes,^38^ and triglycerides (TGs), predominantly stored in lipid droplets,^39^ were then profiled in intestinal epithelial scrapings using UPLC-MS/MS (Figure 3A). The PUFA-enriched Western diet led to an increased proportion of ω-3 and ω-6 PUFAs across all lipid classes analyzed when compared with the Western diet without PUFA enrichment (Figures 3B and S3A; Table 1). More specifically, the PUFA-enriched Western diet enriched arachidonic acid (AA, 20:4 ω-6), eicosapentaenoic acid (EPA, 20:5 ω-3), docosapentaenoic acid (DPA, 22:5 ω-3), and DHA (22:6 ω-3) in the epithelial scrapings, partially replacing linoleic acid (18:2 ω-6) and eicosatrienoic acid (20:3 ω-6) in PC, PE, and TG (Figures 3C–3E).

Next, we assessed which of the epithelial-enriched ω-3 PUFAs, EPA (20:5, ω-3), DPA (22:5, ω-3), and DHA (22:6, ω-3), or their precursor stearidonic acid (SDA, 18:4 ω-3),^40^ and the ω-6 PUFA AA (20:4, ω-6), which are contained in the PUFA-enriched Western diet, exhibited RXRα proximity in a Förster resonance energy transfer (FRET) assay.^41,42^ Notably, the ω-3 PUFA SDA and the ω-6 PUFA AA exhibited concentration-dependent close proximity to RXRα in this assay, as observed for the synthetic ligand 9-*cis* retinoic acid and similarly seen for the ω-3 PUFAs DPA and DHA (Figures 3F and S3B–S3D).^26^ By contrast, the saturated fatty acid palmitic acid (PA, 16:0), the monounsaturated fatty acid oleic acid (OA, 18:1), and EPA (20:5, ω-3) displayed a similar proximity to RXRα as a vehicle (Figure 3F). Therefore, we next studied whether ω-3 PUFAs or ω-6 PUFAs induced RXRα transcription factor activity in model epithelium (MODE-K “IEC”) by a luciferase-based RXRα homodimerization activity assay.^43^ We confirmed that the ω-3 PUFA DHA (but not SDA) and the ω-6 PUFA AA induced RXRα activity in intestinal epithelium to a similar extent as 9-*cis* retinoic acid (Figures 3G, 3H, S3E, and S3F). Finally, we assessed transcriptional RXRα activity in PUFA-exposed IECs by the univariate linear model (using CollecTRI on bulk RNA sequencing), which provided direct evidence that the ω-3 PUFA DHA and the ω-6 PUFA AA induced transcriptional RXRα activity in IECs (Figures 3I and S3G). Collectively, these studies demonstrated that specific ω-3 and ω-6 PUFAs, contained in the PUFA-enriched Western diet and accumulating in epithelial fractions, induced RXRα activity in intestinal epithelium.

### Paneth cells translate PUFA excess into enteritis

**Figure 4.**
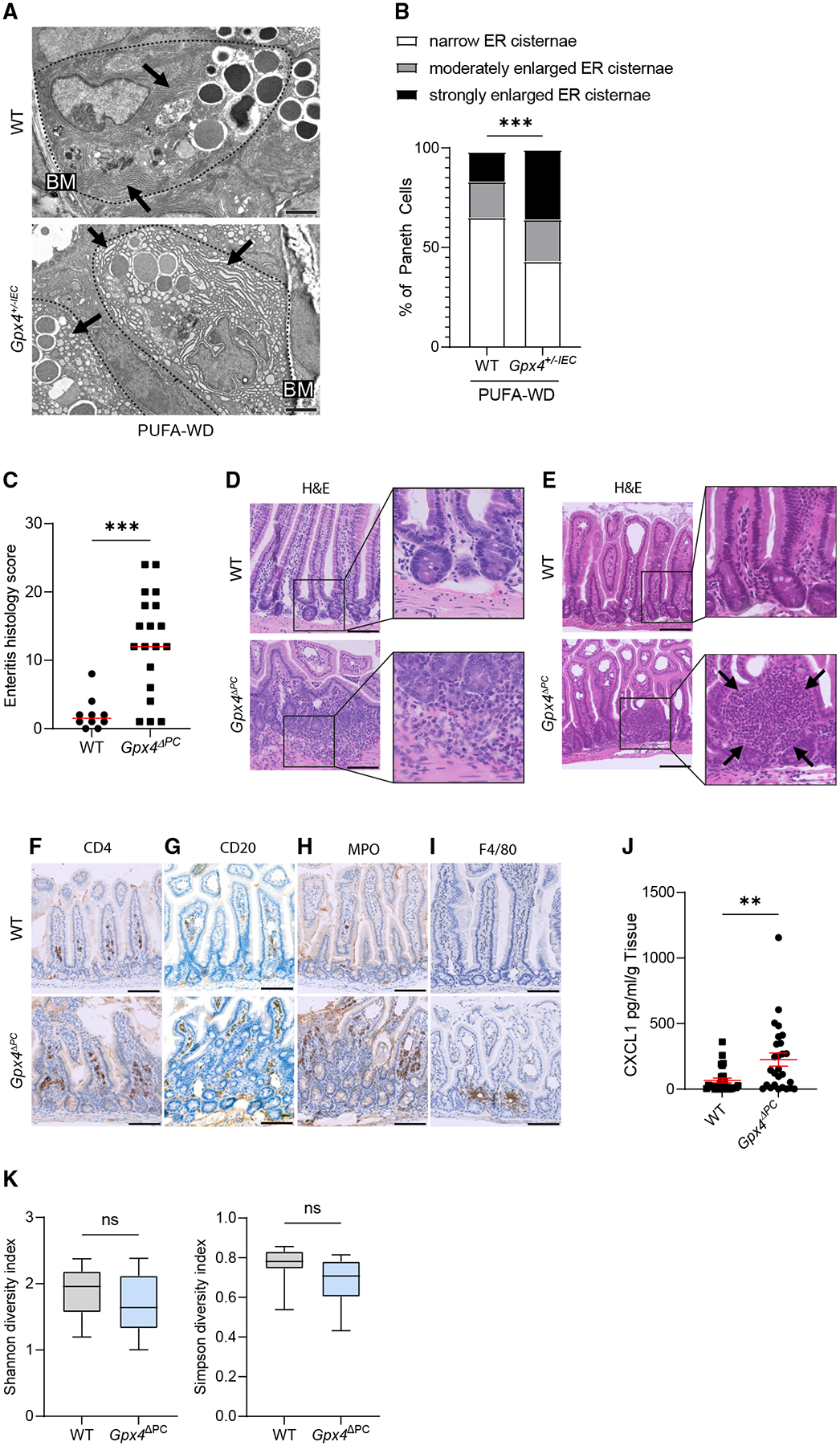
Paneth cells translate PUFA excess into enteritis (A) Representative transmission electron microscopy (TEM) images of Paneth cells (marked by dotted line) from *Gpx4*^+/−*IEC*^ and WT mice after 3-month exposure to a PUFA-enriched Western diet. Note that endoplasmic reticulum (ER) (black arrows) appears dilated in Paneth cells of *Gpx4*^+/−*IEC*^ mice. Scale bars, 2 μm; BM indicates the basement membrane (*n* = 3 mice per genotype). (B) Quantification of Paneth cells showing narrow ER cisternae or moderately or strongly enlarged ER cisternae in TEM images of indicated genotypes after exposure to a PUFA-enriched Western diet (*n* = 3 mice per genotype). (C–E) Enteritis histology score (C) and representative H&E images (D and E) of WT and *Gpx4*^*ΔPC*^ mice exposed to a PUFA-enriched Western diet for 1 month (WT, *n* = 10; *Gpx4*^*ΔPC*^, *n* = 19), and each dot represents an experimental animal. Note the infiltration of immune cells into the bowel wall (D) and a granuloma-like lesion (E) in *Gpx4*^*ΔPC*^ mice. Scale bars, 100 μm. (F–I) Representative images of immunolabeled CD4^+^ T cells (F), CD20^+^ B cells (G), MPO^+^ neutrophils (H), and F4/80^+^ macrophages (I) in WT and *Gpx4*^*ΔPC*^ mice exposed to a PUFA-enriched Western diet for 1 month. Scale bars, 100 μm. (J) Quantification of CXCL1 in supernatant of small intestinal tissue explants by ELISA after 24 h of indicated genotypes exposed to a PUFA-enriched Western diet for 1 month (WT, *n* = 26; *Gpx4*^ΔPC^, *n* = 27). (K) Shannon and Simpson diversity indices of small intestinal stool samples of WT and *Gpx4*^*ΔPC*^ mice exposed to a PUFA-enriched Western diet for 1 month, as assessed by shotgun metagenomics sequencing (WT, *n* = 14; *Gpx4*^ΔPC^, *n* = 6). Data represented as mean ± SEM (unpaired Student’s *t* test) (B, J, and K) and median (Mann-Whitney U test) (C). ns, not significant; **p < 0.01, ***p < 0.001.

Next, we hypothesized that Paneth cells sense the excess of dietary PUFAs to initiate a danger response in the intestine, as suggested by ultrastructural signs of stress in Paneth cells (but not other IEC subtypes) of mice exposed to a PUFA-enriched Western diet (Figures 4A, 4B, and S4A–S4F) and previous experimental findings.^20^ We generated mice lacking *Gpx4* specifically in Paneth cells (i.e., *Defa-6 Cre*^+^;*Gpx4*^*flox/flox*^ or *Gpx4*^*ΔPC*^ mice) (Figure S4G) and exposed these mice (and WT littermates) to a PUFA-enriched Western diet for 1 month. Notably, a PUFA-enriched Western diet induced enteritis in *Gpx4*^*ΔPC*^ mice, but not WT mice, with features of acute and chronic inflammation reminiscent of human CD (Figures 4C–4E). More specifically, a PUFA-enriched Western diet induced patchy enteritis in *Gpx4*^*ΔPC*^ mice, characterized by penetration of the bowel wall and granuloma-like lesions (Figures 4D and 4E) and infiltration of CD4^+^ T cells and CD20^+^ B cells (Figures 4F and 4G). Moreover, infiltration of MPO^+^ neutrophils and F4/80^+^ macrophages in *Gpx4*^*ΔPC*^ mice (Figures 4H and 4I) was paralleled by increased production of the IL-8 homolog CXCL1 (Figure 4J). By contrast, a Western diet without PUFA enrichment or a low-fat control diet did not induce enteritis in *Gpx4*^*ΔPC*^ mice (Figures S4H–S4K).

Paneth cells are known to control gut microbial community structure, which could impact enteritis in our model.^16^ However, we did not observe structural alterations of Paneth cell granules (indicated by lysozyme confocal microscopy) in *Gpx4*^*ΔPC*^ mice when compared with WT littermates on a chow diet or a PUFA-enriched Western diet (Figures S4L–S4O), although a PUFA-enriched Western diet perturbed Paneth cell granule morphology in both genotypes^20^ (Figure S4O). In line with this, metagenomic shotgun sequencing from the small intestinal content of WT and *Gpx4*^*ΔPC*^ mice revealed a comparable α-diversity between *Gpx4*^*ΔPC*^ mice and WT littermates (Figure 4K). Taxonomical and functional profiling also did not reveal significant differences between *Gpx4*^*ΔPC*^ and WT mice exposed to a PUFA-enriched Western diet (Figures S5A–S5C; Tables S3 and S4). Genetic deletion of *Gpx4* specifically in Paneth cells also did not affect the abundance of PAS^+^ goblet cells or DCLK1^+^ tuft cells or the amount of proliferating IECs (labeled by Ki-67) (Figures S5D–S5F). Collectively, these data indicate that Paneth cells rely on GPX4 function as gatekeepers of dietary lipid stress, which appears independent from their exocrine control of gut microbial communities.

### RXRα in Paneth cells mediates PUFA-induced enteritis

**Figure 5.**
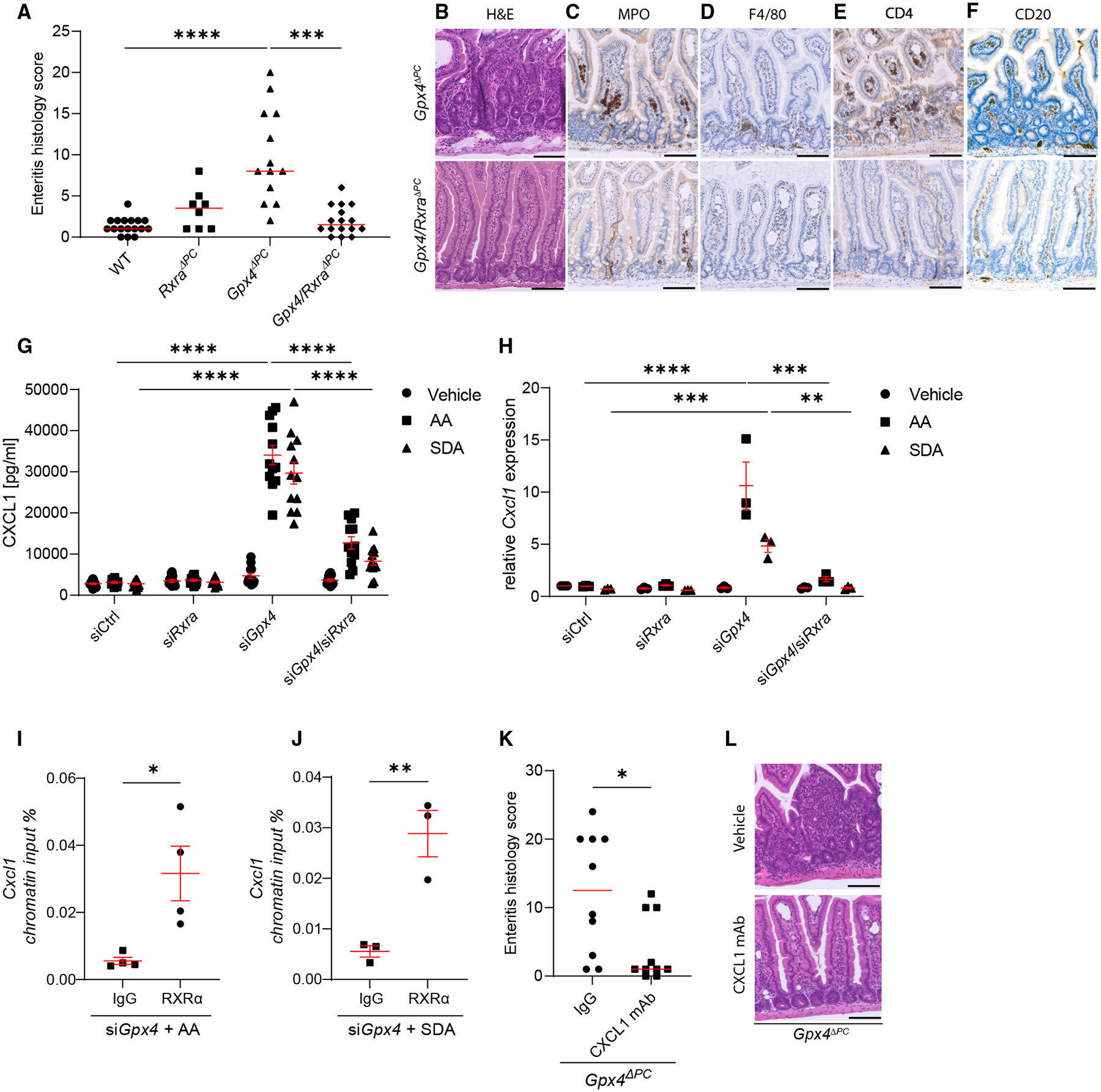
Paneth cell RXRα mediates PUFA-induced enteritis (A and B) Enteritis histology score (A) and representative H&E images (B) of indicated genotypes exposed to a PUFA-enriched Western diet for 1 month (WT, *n* = 17; *Rxrα*^*ΔPC*^, *n* = 8; *Gpx4*^*ΔPC*^, *n* = 13; *Gpx4/Rxrα*^*ΔPC*^, *n* = 16), and each dot represents an experimental animal. Scale bars, 100 μm. (C–F) Representative images of immunolabeled MPO^+^ neutrophils (C), F4/80^+^ macrophages (D), CD4^+^ T cells (E), and CD20^+^ B cells (F) in *Gpx4*^*ΔPC*^ and *Gpx4/Rxrα*^*ΔPC*^ mice exposed to a PUFA-enriched Western diet for 1 month. Scale bars, 100 μm. (G and H) CXCL1 quantification of indicated MODE-K IECs stimulated with vehicle, AA, or SDA for 24 h, as determined in the supernatant by ELISA (G) (*n* ≥ 11) and determined by qPCR after 8 h of stimulation (H) (*n* = 3). (I and J) Chromatin immunoprecipitation indicating a binding of RXRα to the promoter region of CXCL1 in si*Gpx4* IECs stimulated with AA (I) and SDA for 4 h (J) (*n* = 4 for si*Gpx4* AA immunoglobulin G [IgG] and RXRα, *n* = 3 for si*Gpx4* SDA IgG and RXRα). (K and L) Enteritis histology score (K) and representative H&E images (L) of *Gpx4*^*ΔPC*^ mice exposed to a PUFA-enriched Western diet for 1 month and treated with a monoclonal antibody against CXCL1 or IgG control for the last 3 days of the experiment (*n* = 10 each group), and each dot represents an experimental animal. Scale bars, 100 μm. Data represented as median (Kruskal-Wallis test with Dunn’s correction) (A), mean ± SEM (one-way ANOVA with post hoc Bonferroni) (G and H), mean ± SEM (unpaired Student’s *t* test) (I and J), and median (Mann-Whitney U test) (K). *p < 0.05, **p < 0.01, ***p < 0.001, ****p < 0.0001.

Next, we determined whether Paneth cells exploit RXRα to translate PUFA excess into enteritis in our model. We crossed mice that deleted both alleles of *Rxra* in Paneth cells (Figure S6A) onto *Gpx4*^*ΔPC*^ mice (i.e., *Defa-6 Cre*^+^;*Gpx4*^*flox/flox*^*;Rxra* ^*flox/flox*^ mice or *Gpx4/Rxra*^*ΔPC*^) and then exposed these mice (and respective controls) to a PUFA-enriched Western diet for 1 month. Deletion of *Rxra* in Paneth cells abolished PUFA-induced enteritis in *Gpx4/Rxra*^*ΔPC*^ mice as compared with *Gpx4*^*ΔPC*^ mice indicated by enteritis histology scoring (Figures 5A and 5B) and *in situ* immune cell phenotyping (Figures 5C–5F). Gut microbial composition and the related genetic repertoire were comparable in *Gpx4/Rxra*^*ΔPC*^ double-mutant mice and controls after exposure to a PUFA-enriched Western diet (Figures S5A–S5C; Tables S3 and S4), suggesting that endocrine (leukocyte-like) functions of Paneth cells were mediating protection against PUFA-induced enteritis. Indeed, CXCL1 expression in *Gpx4*-deficient epithelium was induced by dietary PUFAs and was transcriptionally mediated by *Rxra* (Figures 5G, 5H, and S6B). Importantly, RXRα heterodimerization partners encoded by *Rara*, *Fxr*, *Lxra*, *Ppara*, *Ppard*, and *Pparg* were dispensable for the control of PUFA-induced CXCL1 production (Figures S6C–S6F), and lipid peroxidation and mitogen-activated protein kinase (MAPK) signaling (previously identified stress pathways driving CXCL1 production and enteritis in this model^37^) were unaffected by *Rxra*-deficiency (Figures S6G and S6H). Moreover, PUFAs did neither elicit an inflammatory response from *Gpx4*-deficient RAW 264.7 macrophages (Figures S7A–S7C) or bone-marrow-derived macrophages (Figures S7D and S7E) nor from *Gpx4*-deficient DC2.4 dendritic cells (Figures S7F and S7G). These studies indicated a specific cellular and molecular context in which dietary PUFAs triggered *Cxcl1* expression in IECs, which was sensed and mediated by RXRα. As RXRα acts as a transcription factor, we studied whether RXRα directly bound to the *Cxcl1* promoter in intestinal epithelium. Using the Ensembl genome browser^44^ and the transcription factor database JASPAR,^45^ we identified a potential RXRα binding site in the *Cxcl1* promoter region, and we conducted chromatin immunoprecipitation after PUFA exposure to confirm that RXRα bound to the *Cxcl1* promoter region in *Gpx4*-deficient epithelium (Figures 5I and 5J). A neutralizing monoclonal CXCL1 antibody^46^ reduced PUFA-induced enteritis in *Gpx4*^*ΔPC*^ mice (Figures 5K and 5L), indicating that RXRα-mediated *Cxcl1* expression is driving enteritis in this model.

Collectively, these data demonstrated that Paneth cell RXRα senses and translates dietary PUFA excess into chronic enteritis reminiscent of CD, partly by governing CXCL1 expression. By contrast, *Rxra* did not affect susceptibility to experimental colitis induced by dextran sulfate sodium (Figures S8A–S8C), and *Rxra* did not control epithelial cytokine production induced by lipopolysaccharide or tumor necrosis factor (Figures S8D and S8E). As such, our study provides experimental evidence for a site-specific inflammatory action of RXRα activity in the context of PUFA exposure in susceptible small intestinal Paneth cells.

### Oral isotretinoin exposure blocks PUFA-induced enteritis and reduces the odds of developing CD

**Figure 6.**
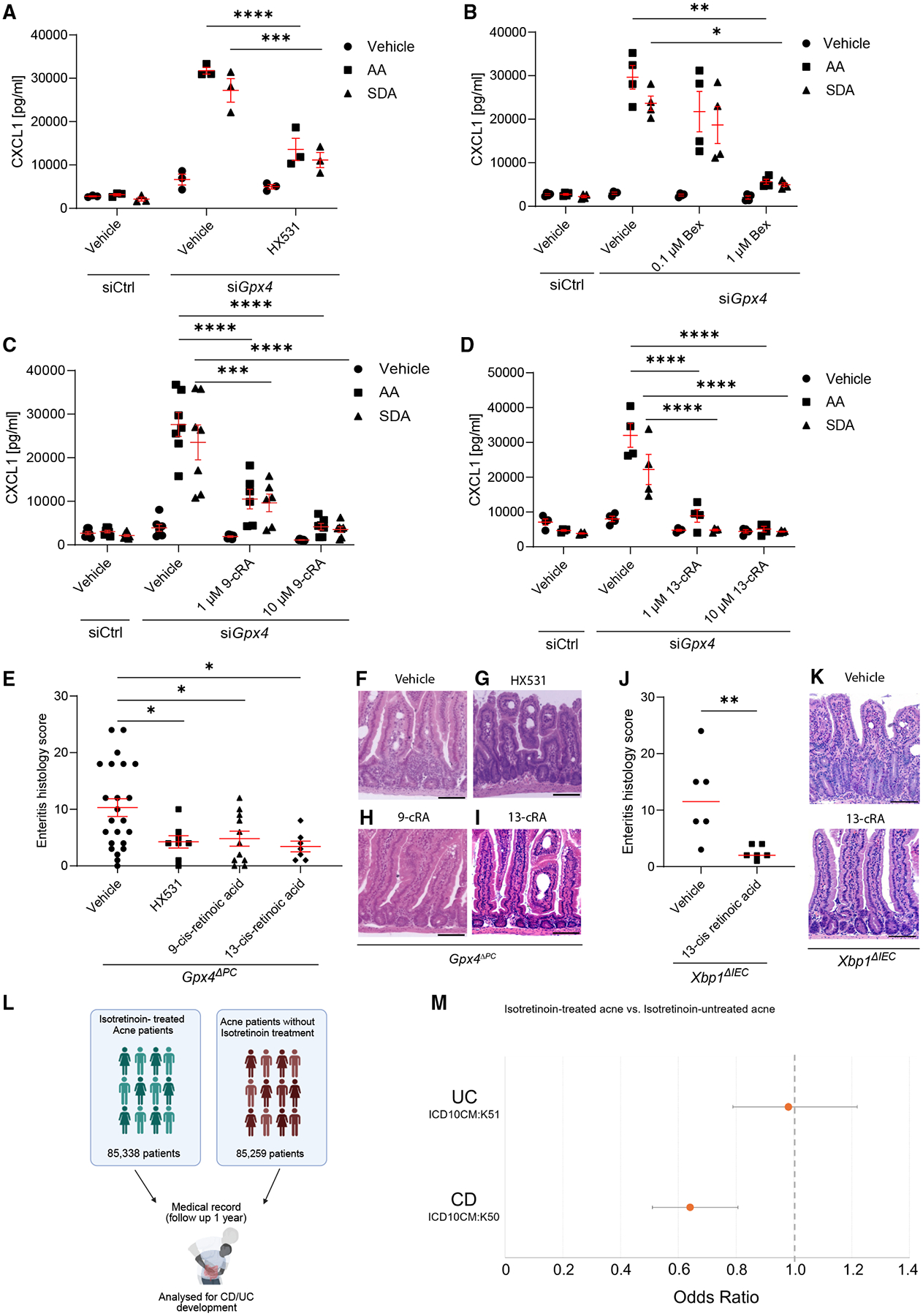
Isotretinoin treatment ameliorates PUFA-induced enteritis and reduces the odds of developing CD (A) CXCL1 quantification of indicated MODE-K IECs stimulated with vehicle, AA, and SDA for 24 h and co-treated with the RXRα antagonist HX531 or vehicle, determined in the supernatant by ELISA (*n* = 3). (B–D) CXCL1 quantification of indicated MODE-K IECs stimulated with vehicle, AA, and SDA for 24 h and co-treated with the RXRα agonists bexarotene (B), 9-*cis* retinoic acid (C), or 13-*cis* retinoic acid (D) compared with vehicle as determined in the supernatant by ELISA (B and D, *n* = 4; C, *n* ≥ 6). (E–I) Enteritis histology score (E) and representative H&E images (F–I) of *Gpx4*^*ΔPC*^ mice exposed to a PUFA-enriched Western diet for 1 month and treated with vehicle (*n* = 23), the RXRα antagonist HX531 (*n* = 8), the RXRα agonist 9-*cis* retinoic acid (*n* = 11), or the RXRα agonist 13-*cis* retinoic acid (*n* = 7) for the last 3 days of the experiment, and each dot represents an experimental animal. Scale bars, 100 μm. (J and K) Enteritis histology score (J) and representative H&E images (K) of *Xpb1*^*ΔIEC*^ mice exposed to a PUFA-enriched Western diet for 1 month and treated with vehicle (*n* = 6) or the RXRα agonist 13-*cis* retinoic acid (*n* = 6) for the last 3 days of the experiment, and each dot represents an experimental animal. Scale bars, 100 μm. (L) Schematic representation of experimental design in TriNetX. 85,338 patients with acne that received oral isotretinoin treatment were compared with 85,259 age- and sex-matched patients with acne that never received isotretinoin treatment. Incidence of CD or UC development was assessed after 1 year in both groups. (M) Forest plot indicating the OR of developing UC and CD in isotretinoin (13-*cis* retinoic acid)-treated and untreated patients with acne after 1 year, as retrospectively assessed by analysis from medical records in TriNetX. Data represented as mean ± SEM (one-way ANOVA with post hoc Bonferroni) (A–E) and median (Mann-Whitney U test) (J). *p < 0.05, **p < 0.01, ***p < 0.001, ****p < 0.0001.

Finally, we studied whether pharmacologic modulation of RXRα activity with chemical agonists or antagonists affected PUFA-induced chemokine production and enteritis in our model and whether this could be relevant for CD. The RXRα antagonists HX531^47^ and LG100754^48^ blocked PUFA-induced CXCL1 production in IECs (Figures 6A and S8F). Notably, also the RXRα agonists bexarotene, 9-*cis* retinoic acid, and its precursor 13-*cis* retinoic acid,^49^ dose-dependently attenuated CXCL1 expression in IECs upon PUFA stimulation, suggesting competitive interactions between PUFAs and retinoic acids with RXRα (Figures 6B–6D, S8G, and S8H), as previously reported.^50^ In line with this, oral exposure to the RXRα antagonist HX531, similar to the RXRα agonist 9-*cis*-retinoic acid and its precursor 13-*cis*-retinoic acid, ameliorated PUFA-induced enteritis in *Gpx4*^*ΔPC*^ mice (Figures 6E–6I). Likewise, oral exposure to 13-*cis*-retinoic acid also blunted PUFA-induced enteritis in an independent model, as seen in mice with intestinal-epithelial-specific deletion of *X-box-binding protein 1* (i.e., *Xbp1*^*ΔIEC*^ mice) exposed to a PUFA-enriched Western diet (Figures 6J and 6K).^37^

In humans, oral 13-*cis* retinoic acid (also called isotretinoin) is used for the systemic treatment of severe acne in adolescents and adults and is partially metabolized to 9-*cis* retinoic acid.^34,51^ This enabled us to test whether isotretinoin therapy affected the odds of developing CD. Specifically, we analyzed electronic medical records from the TriNetX database^52^ and retrospectively assessed whether oral isotretinoin therapy in patients with acne in the United States affected the odds of developing CD later in life when compared with patients with acne not treated with isotretinoin (Figure 6L). Within the US collaborative network, we defined cohorts of patients with acne treated with isotretinoin (*N* = 85,338), which we compared with sex- and age-propensity score-matched patients with acne not treated with isotretinoin (*N* = 85,259). In these two cohorts, we analyzed outcomes for diagnosis of CD after 1 year. Notably, isotretinoin therapy was significantly associated with protection from small and large intestinal CD (odds ratio [OR] 0.641, 95% confidence interval [CI] [0.509, 0.806]), but not from ulcerative colitis (OR 0.981, 95% CI [0.789, 1.219]) (Figure 6M; Tables 2 and 3). Collectively, these findings suggest that modulation of RXRα activity could be used to prevent or treat CD, a concept that warrants validation by controlled clinical trials.

## DISCUSSION

Western dietary cues exert inflammatory actions in and beyond the gut, and experimental evidence for a metabolic control of chronic gut inflammation in IBD is emerging.^2,12,20^ How long-chain fatty acids, typically enriched in a Western diet, are sensed in the intestine and whether this controls an inflammatory response contributing to human CD is unknown. By analyzing the transcription factor activity of known long-chain fatty acid receptors in a large CD cohort and validation in two independent datasets,^32,33^ we identified increased transcriptional RXRα activity in intestinal epithelium from patients with small intestinal CD, which is pronounced in overweight and obese patients when compared with patients with a health-associated BMI. In experimental model systems, we identified that specific dietary ω-3 and ω-6 PUFAs (with the best evidence for AA and DHA) accumulate in intestinal epithelial fractions, elicit RXRα activity in small intestinal epithelium, and induce transcriptional RXRα activity in a mouse model of CD-like enteritis. These findings are in line with previous studies demonstrating direct binding of dietary PUFAs to RXRα (i.e., AA and DHA)^23,27^ and a recent study demonstrating that γ-linolenic acid is a potential RXR ligand in cardiomyocytes.^53^ We demonstrate that PUFA-induced RXRα activity is driving chronic enteritis in our model, which is emanating from Paneth cells that exhibit evidence for perturbed endocrine (but not exocrine) function. Specifically, PUFA excess is sensed by RXRα and mediates the expression of the IL-8 homolog CXCL1, which did not require known RXRα-interacting transcription factors, such as *Rara*, *Fxr*, *Lxra*, or *Ppars*. Collectively, our study identifies RXRα as a metabolic hub by which Paneth cells act as gatekeepers of nutritional stress. Moreover, the study expands the cellular and molecular understanding of specialized epithelial functions and specifically Paneth cells in the context of nutrient sensing beyond enteroendocrine epithelium.^54^

Earlier clinical trials demonstrated that 4 g/day ω-3 PUFA supplementation (with EPA and DHA) did not ameliorate the course of CD over 58 weeks.^55^ Here, we demonstrate in a mouse model of CD-like enteritis that ω-3 PUFAs and ω-6 PUFAs induce intestinal epithelial RXRα activity mediating chemokine expression in the context of impaired GPX4 activity, a feature of human CD,^36^ thus challenging the concept that ω-3 PUFAs act anti-inflammatory in this particular disease context. This is supported by our previous experimental finding demonstrating that oral gavage of ω-3 PUFAs induced small intestinal inflammation in *Gpx4*^+/−*IEC*^ mice, to a similar extent as the ω-6 PUFA AA.^37^ Our experimental data rather indicate that pharmacological blockade of PUFA-RXRα interactions, either with an RXRα antagonist or with a possibly competitive RXRα agonist, ameliorates PUFA-induced chemokine production and CD-like enteritis. Our experimental findings suggest that isotretinoin could be used to specifically prevent or treat CD, a concept that is supported by our retrospective analysis of electronic health care data from the US. We highlight that such an approach challenges our perception of isotretinoin use in human IBD,^56–58^ thus warranting carefully designed controlled clinical trials in CD.

### Limitations of the study

Some limitations are notable in our study. We acknowledge that Paneth cell RXRα likely exerts pleiotropic functions, which may not only be dependent on direct sensing of PUFA excess in a Western diet. For example, specific dietary PUFAs exhibited a variable degree of RXRα luciferase activity in IECs, and we did not study other potential RXRα agonists in the diet. Moreover, our study focused on PUFA-induced RXRα activity controlling *Cxcl1* expression during enteritis, but not other functions of RXRα. RXRα is known to control cytokine expression in macrophages during infection,^24,30^ expression of truncated RXRα in macrophages promoted IL-6 expression in colitis-associated cancer,^59^ and the RXRα agonist LG101305 ameliorated toxic colitis and related TNF and IL1β expression in mice.^60^ Moreover, RXRα may control long-chain fatty acid metabolism, which could have an impact on phenotypes in our model.^61,62^ As such, an unbiased survey on how RXRα mediates PUFA-induced enteritis, i.e., by direct ligand-receptor interactions (as studied herein) or by RXRα-mediated inflammatory actions beyond PUFA sensing, may disentangle further regulatory mechanisms of intestinal inflammation. An ultrastructural resolution of RXRα ligand binding in the presence or absence of specific dietary PUFAs and synthetic RXRα agonists or antagonists may further help to understand why PUFA-induced CXCL1 production and enteritis can be ameliorated by both synthetic RXRα agonists and antagonists.

Collectively, our findings complement previous studies that linked Paneth cell dysfunction in the context of genetic risk with CD^17,18,63,64^ and convey several implications. In small intestinal CD, which is clinically distinct from colonic IBD,^65^ epithelial GPX4 deficiency and increased RXRα activity may originate from dietary PUFA excess and possibly other environmental cues or genetic risk.^36,37^ A specific druggable cellular and molecular framework in the small intestine, in which PUFA-induced RXRα activity instigates inflammation emanating from Paneth cells and IECs, may explain differential patient responses to PUFA excess in a Western diet. Consequently, restriction of dietary PUFA excess or pharmacological blockade of RXRα activity could be therapeutically used to prevent or treat small intestinal CD, a concept that requires validation in controlled prospective clinical trials.

## RESOURCE AVAILABILITY

### Lead contact

Requests for further information and resources should be directed to the lead contact, Timon E. Adolph (timon-erik.adolph@i-med.ac.at).

### Materials availability

All reagents generated in this study are available from the lead contact upon request.

### Data and code availability

The bulk and single-cell transcriptomics data from mouse and *in vitro* models generated in this study have been deposited in the Gene Expression Omnibus (GEO) under accession number GSE284139. Lipidomics data are availible at Zenodo: https://doi.org/10.5281/zenodo.17775130. Data from the human validation cohort^32^ (HMP2) are publicly available through the IBDs Multi’omics Database website: https://ibdmdb.org. Human bulk RNA-sequencing data from the IBDome cohort were provided by the IBDome Consortium and are available at https://ibdome.org/. Human single-cell RNA-sequencing data were obtained from Mukherjee et al.^33^ Raw data are contained in Data S1.

This study utilized a range of open-source tools for data analysis; however, no new algorithms or tools were developed. Detailed information about the tools and their respective versions can be found in the STAR Methods section. The codes used in this study are available upon request.

## STAR★METHODS

### EXPERIMENTAL MODEL AND SUBJECT DETAILS

#### Human studies

The design and outcome of the IBDome study was recently reported^67^ and patient characteristics of our study cohort are provided in Table S1. Ethics approval for the IBDome study was granted and is detailed in Horn et al.,^67^ and all patients who provided informed consent were included for analysis of small intestinal CD. Validation of our findings was performed in a previously published bulk RNA sequencing study in patients with small intestinal CD.^32^ Tissue from CD patients for confocal immunofluorescence imaging was collected at the Gastroenterology Outpatient Clinic of the Department of Internal Medicine I, Medical University of Innsbruck. Patients provided informed consent to analyze their clinical and biochemical parameters and samples and the Ethics Committee of the Medical University of Innsbruck gave approval for this study (AN4994).

#### TriNetX

TriNetX, LLC is a global federated research network with access to both inpatient and outpatient electronic medical records, including diagnoses, procedures, medications, laboratory values and genomic information. The data used in this study was collected from the TriNetX Us Collaborative Network, which provided access to anonymized electronic medical records of 116 million patients, from 66 US healthcare organizations (HCOs). Measure of Association Analysis for cohort outcomes were performed on the TriNetX Live Advanced Analytics dashboard with only aggregate results from isotretinoin- treated and untreated acne cohorts being surfaced and returned to the platform. This retrospective study is exempt from informed consent. The data reviewed is a secondary analysis of existing data, does not involve intervention or interaction with human subjects, and is de-identified per the de-identification standard defined in Section §164.514(a) of the HIPAA Privacy Rule. The process by which the data is de-identified is attested to through a formal determination by a qualified expert as defined in Section §164.514(b)(1) of the HIPAA Privacy Rule.

#### Mice

C57BL/6J *Gpx4*^*flox/flox*^ mice were crossed with C57BL/6J *Villin*-Cre^+/−^ mice to obtain *Gpx4*^*flox/wt*^;*Villin-Cre*^+/−^ mice with a deletion of one allele of *Gpx4* in the intestinal epithelium (*Gpx4*^+/−*IEC*^), as previously reported.^36^ C57BL/6J Rxratm1Krc/J (*Rxra*^*flox/flox*^) mice (Jackson Laboratory, #013086) were crossed with C57BL/6J *Villin*-Cre^+/−^ mice to obtain *Rxra*^*flox/flox*^;*Villin-Cre*^+/−^ mice with a deletion of both alleles of *Rxra* in the intestinal epithelium (*Rxra*^*ΔIEC*^). C57BL/6J *Gpx4*^*flox/flox*^ mice were crossed with C57BL/6J *Defa6-Cre* ^+/−^ mice^17^ to obtain *Gpx4*^*flox/flox*^;*Defa6-Cre*^+/−^ mice with targeted *Gpx4* deletion of both alleles specifically in Paneth cells (*Gpx4*^*ΔPC*^). C57BL/6J Rxratm1Krc/J (*Rxra*^*flox/flox*^) mice were crossed with C57BL/6J *Defa6-Cre*^+/−^ mice to obtain *Rxra*^*flox/flox*^; *Defa6-Cre*^+/−^ (*Rxra*^*ΔPC*^) mice. *Gpx4*^*ΔPC*^ mice were crossed with C57BL/6J Rxratm1Krc/J mice (*Rxra*^*flox/flox*^) to obtain *Rxra*^*flox/flox*^;*Gpx4*^*flox/flox*^;*Defa6-Cre*^+/−^ (*Gpx4/Rxra*^*ΔPC*^) mice. C57BL/6J *Gpx4*^*flox/flox*^ mice were crossed with C57BL/6J *LysMcre*^+/−^ mice to obtain *Gpx4*^*flox/wt*^;*LysMcre*^+/−^ mice (termed *Gpx4*^+/− *MDSC*^ mice) with a deletion of one allele of *Gpx4* in myeloid cell lineages. *Xbp1*^*fl/fl*^ *Villin-Cre*^+/−^ C57BL/6J mice (termed *Xbp1*^*ΔIEC*^ mice) were maintained as reported previously^17^. The genotyping of the respective strains was performed with genomic DNA extracted from ear biopsies. All experiments were carried out in accordance with institutional guidelines and approval of the relevant authorities (2021–0.464.171, 2025–0.539.607 and 2025–0.405.710). All experiments were performed with sex- and age-matched 8- to 10-week-old littermates. The mice were randomly assigned to experiment and treatment groups. All mice were co-housed under specific pathogen-free (SPF) conditions in the animal facility (ZVTA) of the Medical University of Innsbruck. Dietary regimens and mouse treatments are detailed below.

#### Cell culture & stimulation

Mouse immortalized small intestinal epithelial cells (MODE-K) were kindly provided by D. Kaiserlian. These IECs were cultured in high-glucose DMEM (Lonza, BE12–604F) with 10% FCS (Biochrome, S0115), 10 mM HEPES (Biochrome, L1613), 1 mM non-essential amino acids (Gibco, 11140–035), 100 U/ml penicillin and 100 μg/ml streptomycin (Biochrome, A2213) at 37°C in 5% CO_2_. Stimulation of MODE-K IECs was performed for 24 hours unless otherwise stated.

Mouse BMDMs were isolated by flushing tibiae and femurs of WT and *Gpx4*^+/− *MDSC*^ mice with ice cold PBS. After the lysis of erythrocytes (R&D, WL2000), cells were washed with PBS. The BMDMs were cultured for 6 days with 50 ng/ml recombinant murine M-CSF (Peprotech, 315–02) in high-glucose DMEM (Lonza, BE12–604F) with 10% FCS (Sigma Aldrich, S0615), 100 U/ml penicillin and 100 μg/ml streptomycin (Biochrome, A2213) at 37°C in 5% CO_2_.

Mouse immortalized dendritic cells DC2.4 (Cytion, 305515) were cultured in RPMI 1640 (PanBiotech, P04–18500) with 10% FCS (Sigma Aldrich, S0615), 100 U/ml penicillin and 100 μg/ml streptomycin (Biochrome, A2213) at 37°C in 5% CO_2_.

Mouse immortalized macrophages RAW 264.7 (Cytion, 400319) were cultured in RPMI 1640 (PanBiotech, P04–18500) with 10% FCS (Sigma Aldrich, S0615), 100 U/ml penicillin and 100 μg/ml streptomycin (Biochrome, A2213) at 37°C in 5% CO_2_.

The following reagents were used for cell culture stimulation: arachidonic acid (AA, 20 μM, Sigma Aldrich, A3611), docosahexaenoic acid (DHA, 50 μM, Sigma Aldrich D2534), stearidonic acid (SDA, 50 μM, Sigma Aldrich SMB00291) mTNF-α (50 ng/ml, PeproTech, 315–01A), LPS (100 ng/ml, Sigma Aldrich, L4524), 9-cis retinoic acid (1–10 μM, Sigma Aldrich, R4643), LG100754 (5μM, Sigma Aldrich, SML0771), HX531 (10μM, Tocris, 3912), Bexarotene (0.1–1 μM, Sigma Aldrich, 200499), 13-cis retinoic acid (1–10 μM Sigma Aldrich, R3255).

### METHOD DETAILS

#### Dietary regimen

Eight- to 10-week-old WT, *Gpx4*^+/−*IEC*^, *Gpx4*^*ΔPC*^, *Rxra*^*ΔPC*^, *Xbp1*^*ΔIEC*^ and *Gpx4/Rxra*^*ΔPC*^ mice were fed a PUFA-enriched Western diet (PUFA WD, ssniff, TD88137 + 10% fish oil) for three months or 4 weeks ad libitum. A low-fat diet (CD88137) and a Western diet (TD88137) served as controls. The dietary PUFA composition is detailed in Table S5.

#### Mouse treatment

HX531 (Tocris, Cat. No. 3912) at a dose of 50 mg/kg bodyweight or vehicle was administered via oral gavage twice daily and 9-cis-retinoic acid (Sigma Aldrich, R4643) at a dose of 50 mg/kg bodyweight or vehicle or 13-cis-retinoic acid (Sigma Aldrich, R3255) at a dose of 30 mg/kg bodyweight or vehicle was administered via oral gavage daily and anti-CXCL1 antibody (R&D Systems, MAB4531) at a dose of 100μg/100μl/mouse/day or IgG (R&D Systems, AB-105-C) was administered once daily via intraperitoneal injection to WT and *Gpx4*^*ΔPC*^ mice. Animals were fed a PUFA-enriched WD for 4 weeks ad libitum and received oral gavage or intraperitoneal injections of the respective substance (or vehicle) for the last 3 days of the experiment. The mice were sacrificed and samples were immediately collected six hours after the final administration of the respective substances. Co-housed WT and *Rxra*^*ΔIEC*^ mice were treated with 2,5% Dextran sulfate sodium (DSS, MP Biomedicals, 160110) given in drinking water ad libitum for five consecutive days which was followed by tap water until experimental closure.

#### Histology

The collected intestinal tissue was fixed in formalin immediately after sacrifice. Afterwards the tissue was dehydrated, paraffin-embedded and sectioned. Accordingly, the tissue was stained with hematoxylin & eosin as previously described.^37^ The assessment of enteritis and colitis severity was performed by a blinded expert pathologist using a composite scoring system, consisting of five histological subscores, as previously described.^17,36,37^

#### Immunofluorescence

Intestinal sections were deparaffinized using xylene and rehydrated afterwards by descending-gradient ethanol. Heat-mediated antigen-retrieval was performed with citrate buffer (Vector laboratories, H-3300–250) for 12 min at sub-boiling temperature. The slides were then washed and blocked (Protein Block, serum-free, Dako, X090930–2) for 30 min at RT and incubated o/n with the primary antibody diluted in REAL Ab Diluent (Dako, S202230-2) at 4 °C. Slides were then washed three times in PBS before incubating with Alexa Fluor 488 (Invitrogen, A-11008) or Alexa Fluor 555 (Invitrogen, A-31572) secondary antibody for one hour at RT. Slides were washed with PBS and afterwards mounted with Prolong Diamond Antifade reagent with DAPI (Invitrogen, P36962). Imaging was performed with an Axio Observer Z1 confocal microscope (Carl Zeiss), slides were analyzed with Zen 2012 and ImageJ software. Following antibodies were used: anti-RXRα (1:50; CST, 3085), anti-Lysozyme (1:200; Abcam, ab108508), anti-GPX4 (1:400, Abcam, ab125066).

#### Immunohistochemistry and PAS labeling

Intestinal sections were deparaffinized using xylene and rehydrated afterwards by descending-gradient ethanol. Heat-mediated antigen retrieval was performed with citrate buffer (Vector laboratories, H-3300–250) for 12 min at sub-boiling temperature. The slides were then washed and blocked (Protein Block, serum-free, Dako, X090930–2) for 30 min at RT and incubated at 4 °C o/n with the primary antibody diluted in REAL Ab Diluent (Dako, S202230-2). Slides were then washed three times using PBS and incubated with a horseradish peroxidase (HRP)-labelled secondary antibody for 30 min at RT. Immunoreactivity was visualized by HRP-driven 3,3 diaminobenzidine (DAB) turnover. PAS labeling was performed following the manufacturer’s Guidelines. Following antibodies were used: anti-F4/80 (CST, 70076), anti-MPO (Cell Marque, 289A), anti-CD4 (Roche, 05552737001), anti-CD20 (Dako, M0755), anti-DCLK1/DCAMKL1 (CST, 62257), anti-Ki67 (Roche, 05278384001).

#### Electron microscopy

Small intestinal mouse tissues were processed by standard methods as previously described.^68^ Thin epoxy resin sections were observed with a Philips CM120 transmission electron microscope. Quantification of Paneth cells with strong dilatation of the ER served as indication of cellular stress (whereas moderate ER dilation often reflects higher metabolic activity and may occur in healthy tissues). Paneth cells with different degrees of ER dilatation were manually counted on digital micrographs by a TEM expert blinded to sample identity from 3 mice per genotype and condition.

#### siRNA silencing

MODE-K IECs were silenced according to the manufacturer’s recommendations for 48 hours unless otherwise stated. The following siRNAs were used for siRNA silencing of MODE-K IECs: *Gpx4* siRNA (Ambion, s122098), *Rxra* siRNA (Ambion, s73216), *Lxra* siRNA (Ambion, s75783), *Fxr* siRNA (Ambion, s73231) *Ppara* siRNA (Ambion s72005), *Pparg* siRNA (Ambion s72013), *Ppard* siRNA (Ambion s72011) together with RNAiMAX (Thermo Fisher Scientific, 13778150).

#### Whole tissue culture

For whole tissue culture experiments, the small intestine of the respective mice fed a PUFA-enriched Western diet for 4 weeks was cleansed of adhering stool/tissue by flushing with ice cold PBS. A 5mm whole tissue piece was removed from the small intestine and flushed with antibiotic and antifungal cocktail containing 100 μg/ml streptomycin, 100 U/ml penicillin, 10 μg/ml Fluconazole and 0,15 mM Metronidazole to remove intestinal microbiome. The tissue pieces were weight and cultured in in high-glucose DMEM (Lonza, BE12–604F) with 10% FCS (Biochrome, S0115), 10 mM HEPES (Biochrome, L1613), 1 mM non-essential amino acids (Gibco, 11140–035), 100 U/ml penicillin and 100 μg/ml streptomycin (Biochrome, A2213) at 37°C in 5% CO_2_ for 24h.

#### RNA extraction and qRT-PCR

RNA was isolated from MODE-K cells using the RNeasy mini kit (Qiagen, 74104) according to the manufacturer’s recommendations. For cDNA synthesis, M-MLV reverse transcriptase (Invitrogen, 28025013) was used. qRT-PCR was performed on a C1000 Touch (BioRad) with a GoTaq qPCR Master Mix (Promega, A6001). The primers sequences are listed in Table S6.

#### Lipid peroxidation detection by flow cytometry

For the detection of lipid peroxidation in MODE-K IECs, cells were labelled with BODIPY581/591 C11 (Invitrogen, D3861). MODE-K IECs were incubated for 30 min at 37 °C with BODIPY581/591 C11 in FACS buffer. Cells were then washed with PBS, resuspended in FACS buffer, filtered through a 40 μm strainer and analyzed on a Cytoflex S (Beckman Coulter). To exclude dead cells, DAPI was used.

#### Chromatin immunoprecipitation

For Chromatin immunoprecipitation SimpleChIP Enzymatic Chromatin IP Kit (Magnetic Beads) (CST, 9003) was used according to manufacturer’s recommendations. Briefly, cells were crosslinked with 1% formalin for 10 minutes. Afterwards, chromatin was fragmented via enzymatic digestion using micrococcal nuclease. Chromatin was immunoprecipitated overnight at 4°C with anti-RXRα (CST, 3085), while rabbit IgG (CST, 2729) served as a control. Antibody-antigen complexes then were captured using Protein G magnetic beads. After chromatin crosslink removal, DNA was purified using DNA purification spin columns. Purified DNA was then analyzed by CHIP-qPCR. Primers amplifying a potential RXRα binding site in the CXCL1 promoter region according to the Ensembl genome browser release 106 (GRCm39) and JASPAR transcription factor database were used. The amount of immunoprecipitated target DNA is represented as a percentage of input chromatin. Primers used for qPCR were as follows: F 5’-TTGACCCTGAAGCTCCCTTGG and R 5’- CGTTCAGGGGTCATATGCCAG.

#### RXRα reporter assay

We used MODE-K cells which we stably transfected with a lentiviral construct that expresses Firefly-Luciferase under control of a RXRE promoter. The Lentivirus was purchased from LipExoGen (LTV-0047–3S) and transduction was performed according to the manufacturer’s specifications. In brief, we incubated 5×10 ^5 cells with virus in a 6-well culture plate and titrated the virus so that transduction resulted in 5–10 resistant colonies after puromycin selection. The cells created in this way were expanded and used in the following experiments. Cells were seeded in white walled 96-well cell culture plates (Corning, 3610), silenced for 48h and stimulated with polyunsaturated fatty acids and a positive and negative control for 24h. ONE-Glo^™^ Luciferase Assay System (Promega, E6110) was used according to manufacturer’s recommendations for luminescence quantification. Briefly, 100μl of reagent was added to MODE-K IECs grown in 100μl of cell culture medium after 24h of stimulation. Luminescence was measured using an Infinite PRO 200 (Tecan) 20 minutes after addition of reagent at room temperature.

#### Immunoblot

Proteins were extracted from MODE-K IECs using M-PER Protein Extraction Reagent (Thermo Fisher Scientific, 78501) with protease and phosphatase inhibitors (Thermo Fisher Scientific, 78443). We determined the protein concentration by Bradford Assay (Bio-Rad Laboratories, 5000006). Afterwards, an equal amount of protein was denatured using Laemmli Buffer at 95°C for 5 min, loaded onto SDS-PAGE and transferred to polyvinylidene fluoride membranes (GE healthcare, GE10600023). The membranes were then blocked in 5% skim milk and incubated o/n at 4°C with the primary antibody. For visualization of the signals, we used a HRP-conjugated secondary antibody (CST, 7074) and ECL Select Western Blotting Detection Reagent (Amersham, RPN2235). The following antibodies were used for immunoblotting: Anti-GPX4 (Abcam, ab125066), anti-phospho-SAPK/JNK (CST, 4668), anti-phospho-c-Jun (CST, 2361), anti-SAPK/JNK (CST, 9252), anti-c-Jun (CST, 9165), anti-phospho-IRE1α (Abcam, ab48187), anti-IRE1α (CST, 3294), anti-GAPDH (CST, 2118) and anti-RXRα (CST, 3085).

#### Enzyme-linked immunosorbent assay

For the quantification of CXCL1 and IL-6 in supernatant, enzyme-linked immunosorbent assay (ELISA) was performed. The samples were collected as follows: supernatants from MODE-K IECs, whole tissue cultures, RAW 264.7 macrophages, murine bone marrow derived macrophages or DC2.4 dendritic cells were collected, centrifuged at 300 g for 5 min and stored at −20 °C. The assays were performed according to the manufacturer’s recommendations. The following ELISAs were used: murine CXCL1/KC (R&D, DY453), murine IL-6 (BD Bioscience, 555240).

#### TR-FRET RXR alpha assay

The TR-FRET assay was performed using the LanthaScreen TR-FRET RXR alpha Coactivator Assay Kit, goat (Invitrogen, PV4797) according to the manufacturers protocol. Following concentrations were used as recommended by the manufacturer: Fluorescein-PGC1α 500 nM, TB anti-GST antibody 5 nM, RXR alpha LBD-GST 10 nM, DTT 5mM, and 10 μl of the testing compounds. We analyzed 20 μl of the reaction mixture after 3 hours incubation at RT using an Infinite PRO 200 (Tecan), with excitation at 340 nm (30 nm bandwidth) and the emission filter at 520 nm (25 nm bandwidth) and 495 nm (10 nm bandwidth). The TR-FRET ratio was calculated by dividing the emission intensity at 520 nm by the corresponding signal at 495 nm. Following test compounds were used: palmitic acid (Sigma Aldrich, P0500), oleic acid (Sigma Aldrich, O1383), arachidonic acid (Sigma Aldrich A3611), stearidonic acid (Sigma Aldrich, SMB00291), eicosapentaenoic acid (Sigma Aldrich, E2011), docosapentaenoic acid (Sigma aldrich, 43002), docosahexaenoic acid (Sigma Aldrich, D2534) and 9-cis retinoic acid (Sigma Aldrich, R4643).

#### Bulk RNA-sequencing of MODE-K IECs

After stimulation at 37°C for 8h, cell culture medium was discarded and cells were rinsed once with PBS. RNA isolation was performed according to manufacturer’s instructions using a RNeasy mini kit (Qiagen, 74104). Purified RNA was stored at −80 °C. Purified total-RNA was submitted for transcriptome analysis at the Medical University Innsbruck Core facility, using the QuantSeq 3′ mRNA-Seq Library Prep method (Lexogen, Vienna Biocenter). Quality-validated, barcoded libraries were multiplexed and sequenced using Illumina NovaSeq technology.

#### Single-cell preparation and scRNA-sequencing

Mice were fed a PUFA-enriched Western diet for 12 weeks. For single cell isolation, the small intestine (SI) was cleaned of adhering stool/tissue. Afterwards the middle 60 % of the SI was flushed with ice cold PBS. The SI was then opened longitudinally and washed again with ice cold PBS. Thereafter, the tissue was cut into small pieces and digested in 50ml PBS containing 2mM EDTA. Samples were shaken regularly for the next 30min. The supernatant was collected and remaining tissue pieces were dissolved in ice cold PBS and shaken again. This step was repeated 4 times. Supernatant was then centrifuged for 5 min at 300 g at 4°C and the resulting cell pellet was dissolved in trypsin + PBS (1:1), vortexed gently and dissolved in DMEM containing FCS. Accordingly, cells were counted and incubated with beads for live dead separation using a dead cell removal kit (Miltenyi Biotec, 130-090-101) according to the manufacturer’s recommendations. Briefly, the cell pellets were dissolved in 100 μl of Micro Beads Solution and then incubated at RT for 15 min. Dead cells were retained in MACS Columns (Miltenyi Biotec) during separation with a magnetic MACS separator (Miltenyi Biotec). The viable cells were collected and centrifuged at 300 g and 4°C for 5 min. Pellets were dissolved in 100 μl ice-cold PBS containing 0,4 % BSA and transferred on ice to the Medical University of Innsbruck MultiOmics Sequencing Core facility. Adequate Cell integrity and concentration was verified with trypan blue staining (Sigma, Cat# T8154) and by counting with a hemocytometer (Marienfeld Neubauer, Cat# 0640010). Final cell suspensions were processed with a Chromium single cell controller (10xGenomics) and Chromium Next GEM Single Cell 3’ Kit v3.1 chemistry, targeting 8000 cells per sample. The resulting libraries were multiplexed and sequenced with Illumina NovaSeq S4 flowcell technology, generating in total approximately 2.5 B read pairs.

#### Bioinformatic analysis

##### Mouse Single-Cell Data Pre-Processing, Quality Control and data analysis

The bioinformatic pre-processing of 10x fastq sequencing files was performed with cellranger v7.1.0 (10x Genomics) using the nf-core scRNA-seq pipeline v2.4.1^66^ with the GRCm39 reference genome and GENCODE vM33 annotations. Raw count matrices were imported into AnnData^69^ and processed with scverse tools.^70^ Ambient RNA was removed using scAR,^71^ and doublets were identified and removed using SOLO,^72^ both implemented in scvi-tools.^73^ Quality control on the denoised counts was done using scanpy,^74^ retaining cells with (1) >1000 transcripts, (2) >500 genes, and (3) <10% mitochondrial transcripts. The top 2000 highly variable genes (HVGs) were selected using scanpy’s “highly_variable_genes” function with flavor=“seurat_v3” and batch_key=“sample”. Cell transcriptomes were embedded into a batch-corrected low-dimensional latent space using scVI,^73,75^ treating each sample as a batch. The neighborhood graph and UMAP embedding^76^ were computed based on the scVI latent space. Cell types were annotated through unsupervised clustering with the Leiden algorithm^77^ and known marker genes (Figure S2A). DESeq2^78^ (version 1.40.2) was used on pseudo-bulk samples for differential gene expression testing. For each cell type and sample, we summed up transcript counts for each gene that is expressed in at least 5% of cells using decoupler-py^79^ (version 1.5.1). P-values were adjusted for multiple hypothesis testing with independent hypothesis weighting (IHW).^80^ Transcription factor analysis was performed with CollecTRI^35^ using a univariate linear model and pathway analysis with PROGENy^81^ using a multivariate linear model as implemented in decoupler-py. As input we used stat values from the DESeq2 analysis.

##### Bulk-RNA sequencing data analysis of MODE-K cells

The nf-core/rnaseq (version 3.8.1)^66^ pipeline was used to align the raw reads to the mouse genome (GRCm39) with STAR^82^ and to assess the read counts on the gene models from GENCODE version M27 with Salmon.^83^ The pipeline was executed with the default parameters except for the gene quantification with Salmon where the following parameter ‘–noLengthCorrection’, which accounts for the Lexogen 3’ QuantSeq RNA sequencing library, was set. Differential expressed genes between si*Gpx4* and siCtrl MODE-K IECs were calculated using DESeq2 (version 1.34.0)^78^ using a fold change threshold of 2 and a FDR of 0.1 after Independent Hypothesis Weighting (IHW).^80^ For transcription factor analysis, a univariate linear model was applied using the R package decoupleR^79^ in conjunction with CollecTRI.^35^ The differential transcription factor activities were plotted as sorted (high to low) barplot. Activities were defined as significantly activated or suppressed based on an FDR threshold of less than 0.1.

##### Analysis of human datasets

Single-cell RNA-sequencing data were provided as cell-type specific Seurat objects from Mukherjee et al.^33^ containing a total of 223,755 cells. The different objects were combined using Seurat v.5.0.3, and the dataset was subsetted for cells from normal and inflamed non-strictured samples, excluding one outlier sample, resulting in 114,093 remaining cells. Pseudo-bulk profiles were then generated in Python with decoupler v.1.4.0, and differential expression analysis between inflamed CD and nonIBD samples performed for each cell type using pydeseq2 v.0.3.4. Transcription factor activity was inferred from the Wald statistics of the DE analysis output in line with the bulk data analysis. The results were visualized as a heatmap with the seaborn module v.0.12.2.

Human bulk RNA-sequencing fastq-files from the IBDome cohort were preprocessed with the nf-core RNA-seq pipeline version 3.4.^66^ In brief, sequencing reads were aligned to the hg38/GRCh38 reference genome with GENCODE v33 annotations using STAR v2.7.7a.^82^ Read counts were quantified with Salmon.^83^ Processed data and annotation tables from the human validation cohort HMP2^32^ were downloaded from https://ibdmdb.org and imported into R v.4.3.2. Samples were filtered for Crohn’s disease and nonIBD and biopsy locations ileum or terminal ileum. Two samples were excluded due to a low number of total counts (<1000). A total of 41 CD and 20 nonIBD samples were used for downstream analyses.

Differential expression analysis was conducted using DESeq2 v1.30.0.^78^ False discovery rates (FDRs) were calculated using IHW.^80^ Genes were considered significantly regulated if they had an absolute log2 fold change (|log2FC|) greater than 1 and an FDR less than 0.1. Volcano plots were generated using the EnhancedVolcano package v1.8.0, and heatmaps were created with the ComplexHeatmap package v.2.18.0. Transcription factor activities were inferred from the Wald statistics of the differential expression analysis output using decoupleR,^79^ employing a univariate linear model and the CollecTRI database.^35^ Activities were defined as significantly activated or suppressed based on an FDR threshold of less than 0.1.

#### Shotgun metagenomics

DNA was isolated from small intestinal fecal material using the DNeasy PowerSoil Pro Kit (Qiagen, 47014) following the manufacturer’s protocol. Extracted DNA was eluted from the spin filter silica membrane with 100 μl of elution buffer and stored at −80 °C. Shotgun metagenomic sequencing of the fecal DNAs was performed at the Competence Centre for Genomic Analysis (Kiel). DNA libraries were generated using the Illumina DNA Prep kit following the manufacturer’s instructions. Libraries were then pooled and sequenced on an Illumina NovaSeq 6000 with 2 × 150 bp.

##### Metagenome data processing

Shot-gun reads were processed using an in-house established workflow from our institute (https://github.com/ikmb/TOFU-MAaPO) that relies on BBtools and bowtie2 for mapping and host decontamination, and bioBakery tools (MetaPhlAn 4.0 and HUMaN 3.6) for taxonomic and functional potential profiling. Raw reads were quality trimmed and mapped to the mouse genome (GRCm39) to discern between sequenced reads from the host and the gut microbiome bacteria.

##### Taxonomic and Functional Potential Analysis

Taxonomic features and quantification of microbial communities’ relative abundances on the 20 samples were done by using MetaPhlAn 4.01^84^ with default parameters and the custom SGB database. MetaPhlAn 4.0 relies on unique marker genes of 26,970 species-level genome bins (SGBs) from a collection of highly curated 1.01M prokaryotic and metagenome-assembled genomes (MAGs). Likewise, functional potential profiling (MetaCyc pathways) in the same samples was computed using HUMAnN 3.6,^85^ with default parameters. All the abundances were transformed to CPMs before any statistical analysis.

##### Statistics for shotgun sequencing and metagenomics

Most of the univariate and multivariate analyses were done in the R statistical software (v.4.2.1), vegan(v.2.6–2),^86^ and MAasLin2 (v.1.10.0).^87^ Within sample diversity (alpha diversity) was explored by computing different diversity indexes (Shannon and Simpson) on species abundance data and finding differences among certain groups (Wilcoxon signed-rank test). Between-sample diversity (beta diversity) and the differences between genotype groups were explored and visualized in a Principal Coordinate Analysis (PCoA) plot. It was quantified as Bray-Curtis distances from Species abundance data. Associations of microbiome composition to different genotypes were tested with the implementation of PERMANOVA models (using adonis2 function from the vegan package). The P and R2 values were determined by 10,000 permutations using genotype as covariable in the model. To detect differences in changes in microbial features, taxonomic or pathways (shot-gun sequencing), between the genotype group over time, we built linear mixed models in the MaAslin2 package that included genotype and sex as fixed effects Feature ~ +genotype+sex). P values were corrected for multiple hypothesis testing using the Benjamin-Hochberg procedure, and a false discovery rate < 0.05 was defined as the significant threshold.

#### Extraction and analysis of phospholipids and triglycerides by UPLC-MS/MS

Lipids were extracted from tissue homogenates of small intestinal scrapings of *Gpx4*^+/−*IEC*^ mice fed a PUFA-WD or a WD for 3 months, by the sequential addition of PBS pH 7.4, methanol (spiked with internal standards), chloroform, and saline (final ratio 14:34:35:17).^88^ The chloroform layer was recovered and evaporated using an Eppendorf Concentrator Plus system (Hamburg, Germany; non-polar phase: high vapor pressure application mode), stored at −20°C, and dissolved in methanol prior to ultra-high-performance liquid chromatography-mass spectrometry (UPLC-MS/MS) analysis. 1-pentadecanoyl-2-oleoyl(d7)-sn-glycero-3-phosphocholine [PC(15:0/18:1-d7), Avanti Polar Lipids, Alabaster, AL/Sigma-Aldrich, Darmstadt, Germany], 1-pentadecanoyl-2-oleoyl(d7)-sn-glycero-3-phosphoethanolamine [PE(15:0/18:1-d7), Avanti Polar Lipids/Sigma-Aldrich] and 1,3-dipentadecanoyl-2-oleyol(d7)-glycerol [TG(15:0/18:1-d7/15:0), Avanti Polar Lipids/Sigma-Aldrich] were used as internal standards.

Lipids were separated on an Acquity UPLC BEH C8 column (130 Å, 1.7 μm, 2.1×100 mm, Waters, Milford, MA) using an ExionLC AD UHPLC system (Sciex, Framingham, MA). For the analysis of phosphatidylcholine (PC) and phosphatidylethanolamine (PE), the gradient containing mobile phase A (water/acetonitrile 90/10, 2 mM ammonium acetate) and B (water/acetonitrile 5/95, 2 mM ammonium acetate) was ramped from 75 to 85% B over 5 minutes and then further increased to 100% B within 2 minutes, which was maintained isocratically for another 2 minutes.^89^ For the analysis of triglycerides (TG), mobile phase A (isopropanol) and mobile phase B (acetonitrile/water 95/5, 10 mM ammonium acetate) were ramped from 90% to 70% B over 6 min followed by isocratic elution for 4 min. The flow rate was set to 0.75 ml/minute and the column temperature to 45°C.

Lipids were detected by a QTRAP6500^+^ mass spectrometer (Sciex) equipped with an IonDrive Turbo V Ion Source and a TurboIonSpray probe for electrospray ionization. The QTRAP6500^+^ system was operated in negative (for PC, PE)^90^ or positive (for TG) ionization mode using scheduled multiple reaction monitoring (MRM).^91^ For the detection of PE and PC species, the transitions from [M-H]^−^ to both fatty acid anions were monitored. For the detection of TG species, the transitions from [M + NH_4_]^+^ adduct ions to [M - fatty acid anion]^+^ ions were monitored, without differentiating between fatty acid positional isomers. Table S7 shows the previously reported optimized mass spectrometry parameters for PC,^92^ PE^89^ and TG.^93^

The instruments were operated by Analyst 1.7.1 (Sciex), and the mass spectra were processed by Analyst 1.6.3 (Sciex). For the quantitative analysis of PC, PE and TG species, combined peak areas from the transitions to the two fatty acid anions (for PC, PE) or the three [M - fatty acid anion]+ ions (for TG) were calculated. Fatty acid compositions are given as percentage of all species detected in the corresponding PE, PC or TG subclass (= 100%).

### QUANTIFICATION AND STATISTICAL ANALYSIS

#### Statistical analysis

Data are represented as mean ± standard error of the mean (SEM) unless otherwise stated. Statistical analysis was performed with GraphPad Prism 9.5.1 and 10.5.0. Outlier testing was performed using a Grubbs test in parametric sample sets. If not otherwise stated, statistical significance was assessed with an unpaired two-tailed Students t-test, a Mann-Whitney U test, a one-way ANOVA with Bonferroni correction or a Kruskal-Wallis test with Dunn’s correction (as appropriate). The sample size (n) is indicated in the figure legends for each experiment. ns not significant; *p <0.05; **p < 0.01; ***p < 0.001; ****p < 0.0001.

## Supplementary Material

Document S1. Figures S1–S8, Tables S5–S7, and TRR241 IBDome Consortium members

Table S1. Patient characteristics of the “IBDome” cohort, related to Figure 1

Table S2. RXRα target genes in the small intestinal mucosa of CD patients compared with non-IBD controls in the “IBDome” cohort, related to Figure 1

Table S3. MAasLin2 output of differential abundance analysis testing at the species level, related to Figures 4 and S5

Table S4. MAasLin2 output of differential abundance analysis testing at the pathway level, related to Figures 4 and S5

Data S1. Unprocessed data underlying the display items in the manuscript, related to Figures 1–6 and S1–S8

SUPPLEMENTAL INFORMATION

Supplemental information can be found online at https://doi.org/10.1016/j.cmet.2025.11.008.

## Figures and Tables

**Table 1.** Proportions of the indicated PUFAs within PC, PE, or TG in small intestinal scrapings at the site of inflammation from *Gpx4*^*+/–IEC*^ mice fed a PUFA-enriched Western diet or a Western diet without PUFA enrichment for 3 months

	WD (% total lipids)	PUFA-WD (% total lipids)	Fold-change (PUFA-WD/WD)
18:2 (ω6)_PC	15.14 ± 0.94	9.99 ± 0.89	0.66
20:3 (ω6)_PC	2.51 ± 0.29	1.44 ± 0.09	0.57
20:4 (ω6)_PC	3.30 ± 0.29	5.56 ± 0.33	1.68
20:5 (ω3)_PC	0.05 ± 0.01	1.32 ± 0.10	26.55
22:5 (ω3)_PC	0.06 ± 0.01	0.49 ± 0.02	8.49
22:5 (ω6)_PC	0.09 ± 0.01	0.30 ± 0.03	3.25
22:6 (ω3)_PC	0.51 ± 0.02	4.26 ± 0.16	8.43
18:2 (ω6)_PE	11.87 ± 0.64	5.12 ± 0.49	0.43
20:3 (ω6)_PE	1.93 ± 0.08	1.16 ± 0.07	0.60
20:4 (ω6)_PE	11.21 ± 0.82	11.41 ± 1.14	1.02
20:5 (ω3)_PE	0.09 ± 0.01	1.25 ± 0.09	14.21
22:5 (ω3)_PE	0.21 ± 0.02	1.71 ± 0.06	7.96
22:5 (ω6)_PE	0.19 ± 0.01	1.14 ± 0.07	6.07
22:6 (ω3)_PE	1.56 ± 0.09	11.15 ± 0.57	7.15
18:2 (ω6)_TG	11.88 ± 0.54	8.35 ± 0.32	0.70
20:3 (ω6/9)_TG	0.35 ± 0.01	0.23 ± 0.01	0.67
20:4 (ω6)_TG	0.91 ± 0.07	1.33 ± 0.07	1.46
20:5 (ω3)_TG	0.14 ± 0.01	8.17 ± 0.79	57.67
22:5 (ω3/6)_TG	0.09 ± 0.01	0.74 ± 0.06	8.08
22:6 (ω3)_TG	0.53 ± 0.04	6.15 ± 0.53	11.53

PUFA-WD, PUFA-enriched Western diet; WD, Western diet without PUFA enrichment.

**Table 2.** TriNetX analysis of acne patients with or without isotretinoin treatment and odds for CD after 1 year

Cohort	Patients in cohort	Patients with outcome		Risk
Isotretinoin-treated acne (*N*)	85,338	120		0.001
Non-isotretinoin-treated acne (*N*)	85,259	187		0.002
		95% CI	z	*p*
Risk difference	−0.001	(−0.001, −0.000)	−3.835	0.000
Risk ratio	0.641	(0.510, 0.806)	N/A	N/A
Odds ratio	0.641	(0.509, 0.806)	N/A	N/A

Risk analysis excluding patients with outcome prior to the time window.

**Table 3.** TriNetX analysis of acne patients with or without isotretinoin treatment and odds for UC after 1 year

Cohort	Patients in cohort	Patients with outcome		Risk
Isotretinoin-treated acne (*N*)	85,427	161		0.002
Non-isotretinoin-treated acne (*N*)	85,328	164		0.002
		95% CI	z	*p*
Risk difference	−0.000	(−0.000, 0.000)	−0.177	0.859
Risk ratio	0.981	(0.789, 1.219)	N/A	N/A
Odds ratio	0.981	(0.789, 1.219)	N/A	N/A

Risk analysis excluding patients with outcome prior to the time window.

**Table:** KEY RESOURCES TABLE

REAGENT or RESOURCE	SOURCE	IDENTIFIER
Antibodies		
Rabbit monoclonal anti-RXRα	Cell Signaling Technology	Cat#3085; RRID:AB_11140620
Rabbit monoclonal anti-Lysozyme	Abcam	Cat#ab108508; RRID:AB_10861277
Rabbit monoclonal anti-GPX4	Abcam	Cat#ab125066; RRID:AB_10973901
Rabbit monoclonal anti-F4/80	Cell Signaling Technology	Cat#70076; RRID:AB_2799771
Rabbit IgG	R&D Systems	Cat#AB-105-C; RRID:AB_354266
Rabbit polyclonal anti-MPO	Cell Marque	Cat#289A; RRID:AB_1158661
Rabbit monoclonal anti-CD4	Roche	Cat#05552737001; RRID:AB_2335982
Mouse monoclonal anti-CD20	Dako	Cat#M0755; RRID:AB_2282030
Rabbit monoclonal anti-DCLK1/DCAMKL1	Cell Signaling Technology	Cat#62257; RRID:AB_2799622
Rat monoclonal anti-CXCL1	R&D Systems	Cat#MAB4531; RRID:AB_2261282
Rabbit monoclonal anti-Ki67	Roche	Cat#05278384001; RRID:AB_2631262
Rabbit monoclonal anti-phospho-SAPK/JNK	Cell Signaling Technology	Cat#4668; RRID:AB_823588
Rabbit monoclonal anti-phospho-c-Jun	Cell Signaling Technology	Cat#2361; RRID:AB_490908
Rabbit polyclonal anti-SAPK/JNK	Cell Signaling Technology	Cat#9252; RRID:AB_2250373
Rabbit monoclonal anti-c-Jun	Cell Signaling Technology	Cat#9165; RRID:AB_2130165
Rabbit polyclonal anti-phospho-IRE1α	Abcam	Cat#ab48187; RRID:AB_873899
Rabbit monoclonal anti-IRE1α	Cell Signaling Technology	Cat#3294; RRID:AB_823545
Rabbit IgG	Cell Signaling Technology	Cat#2729; RRID:AB_1031062
Rabbit monoclonal anti-GAPDH	Cell Signaling Technology	Cat#2118; RRID:AB_561053
Goat anti-Rabbit IgG, Alexa Fluor^™^ 488	Invitrogen	Cat#A-11008; RRID:AB_143165
Anti-rabbit IgG, HRP-linked Antibody	Cell Signaling Technology	Cat#7074; RRID:AB_2099233
Donkey anti-Rabbit IgG, Alexa Fluor^™^ 555	Invitrogen	Cat#A-31572; RRID:AB_162543
Bacterial and virus strains		
RXRE/DR1 Reporter Lentivirus	LipExoGen	Cat#LTV-0047-3S
Biological samples		
Intestinal tissue from CD patients	Gastroenterology Outpatient Clinic of the Department of Internal Medicine I, Medical University of Innsbruck	N/A
Chemicals, peptides, and recombinant proteins		
HX531	Tocris	Cat#3912
9-cis-retinoic acid	Sigma Aldrich	Cat#R4643
13-cis-retinoic acid	Sigma Aldrich	Cat#R3255
Dextran sulfate sodium	MP Biomedicals	Cat#160110
citrate buffer	Vector laboratories	Cat#H-3300-250
Protein Block, serum-free	Dako	Cat#X090930-2
REAL Ab Diluent	Dako	Cat#S202230-2
Prolong Diamond Antifade reagent with DAPI	Invitrogen	Cat#P36962
murine M-CSF	Peprotech	Cat#315-02
FBS Superior	Sigma Aldrich	Cat#S0615
Penicillin-Streptomycin	Biochrome	Cat#A2213
high-glucose DMEM	Lonza	Cat#BE12-604F
HEPES	Biochrome	Cat#L1613
non-essential amino acids	Gibco	Cat#11140-035
RPMI 1640	PanBiotech	Cat# P04-18500
arachidonic acid	Sigma Aldrich	Cat#A3611
docosahexaenoic acid	Sigma Aldrich	Cat#D2534
stearidonic acid	Sigma Aldrich	Cat#SMB00291
LPS	Sigma Aldrich	Cat#L4524
LG100754	Sigma Aldrich	Cat#SML0771
Bexarotene	Sigma Aldrich	Cat#200499
RNAiMAX	Thermo Fisher Scientific	Cat#13778150
M-MLV reverse transcriptase	Invitrogen	Cat#28025013
GoTaq qPCR Master Mix	Promega	Cat#A6001
BODIPY581/591 C11	Invitrogen	Cat#D3861
M-PER Protein Extraction Reagent	Thermo Fisher Scientific	Cat#78501
protease and phosphatase inhibitors	Thermo Fisher Scientific	Cat#78443
Bradford reagent	Bio-Rad Laboratories	Cat#5000006
PVDF membrane	GE healthcare	Cat#GE10600023
ECL Select Western Blotting Detection Reagent	Amersham	Cat#RPN2235
palmitic acid	Sigma Aldrich	Cat#P0500
oleic acid	Sigma Aldrich	Cat#O1383
eicosapentaenoic acid	Sigma Aldrich	Cat#E2011
docosapentaenoic acid	Sigma Aldrich	Cat#43002
Critical commercial assays		
RNeasy mini kit	Qiagen	Cat#74104
SimpleChIP Enzymatic Chromatin IP Kit	Cell Signaling Technology	Cat#9003
ONE-Glo Luciferase Assay System	Promega	Cat#E6110
Mouse Erythrocyte Lysing Kit	R&D	Cat#WL2000
murine CXCL1/KC ELISA	R&D	Cat#DY453
murine IL-6 ELISA	BD Bioscience	Cat#555240
dead cell removal kit	Miltenyi Biotec	Cat#130-090-101
DNeasy PowerSoil Pro Kit	Qiagen	Cat#47014
LanthaScreen TR-FRET RXR alpha Coactivator Assay Kit, goat	Invitrogen	Cat#PV4797
Deposited data		
Single-cell RNA sequencing and bulk RNA sequencing data from mouse and in vitro models	This paper	GEO: GSE284139
Lipidomics data	This paper	Zenodo: https://doi.org/10.5281/zenodo.17775130
Tables S1, S2, S3, and S4	This paper	Supplemental Tables
Data from the IBDome cohort	This paper	https://ibdome.org/
Data from the human validation cohort (HMP2)	Lloyd-Price et al.^32^	https://ibdmdb.org.
Human single-cell RNA-sequencing data	Mukherjee et al.^33^	N/A
Source data	This paper	Data S1
Experimental models: Cell lines		
MODE-K cells	Dominique Kaiserlian	N/A
Mouse BMDMs	This paper	N/A
DC2.4 cells	Cytion	Cat#305515
RAW 264.7 cells	Cytion	Cat#400319
Experimental models: Organisms/strains		
Mouse: C57BL/6J Rxratm1Krc/J	The Jackson Laboratory	Cat#013086
Mouse: C57BL/6J Gpx4 flox/flox	Qitao Ran	N/A
Mouse: C57BL/6J Xbp1 flox/flox	Richard S. Blumberg	N/A
Mouse: C57BL/6J Villin-Cre+/−	The Jackson Laboratory	N/A
Mouse: C57BL/6J LysM-Cre+/−	The Jackson Laboratory	N/A
Mouse: C57BL/6J Defa6-Cre+/−	Richard S. Blumberg	N/A
Oligonucleotides		
*Gpx4* siRNA	Ambion	Cat#s122098
*Rxra* siRNA	Ambion	Cat#s73216
*Lxra* siRNA	Ambion	Cat#s75783
*Fxr* siRNA	Ambion	Cat#s73231
*Ppara* siRNA	Ambion	Cat#s72005
*Pparg* siRNA	Ambion	Cat#s72013
*Ppard* siRNA	Ambion	Cat#s72011
Primers for qPCR see Table S6	This paper	N/A
Primer for CHIP qPCR: F 5’-TTGACCCTGAAGCTCCCTTGG and R 5′- CGTTCAGGGGTCATATGCCAG	This paper	N/A
Software and algorithms		
Zen 2012 software	Zeiss	https://www.zeiss.com/
R version 4.3.2	The R Project for Statistical Computing	https://www.r-project.org/
Python version 3.11.4	Python Software Foundation	https://www.python.org/
nf-core RNA-seq pipeline version 3.4 and 3.8.1	Ewels et al.^66^	https://github.com/nf-core/rnaseq
nf-core scRNA-seq pipeline version 2.4.1	Ewels et al.^66^	https://github.com/nf-core/rnaseq
Cell Ranger version 7.1.0	10x Genomics	https://support.10xgenomics.com/single-cell-gene-expression/software/pipelines/latest/what-is-cell-ranger
FlowJo version 10	FlowJo	https://www.flowjo.com
BioRender	BioRender Software	https://www.biorender.com/
ImageJ	NIH	https://imagej.net/ij/
GraphPad Prism 9.5.1 and 10.5.0	GraphPad software	https://www.graphpad.com/
Other		
TriNetX	Palchuk et al.^52^	https://trinetx.com/
PUFA-enriched Western diet	ssniff	Cat#TD88137 + 10% fishoil
low-fat diet	ssniff	Cat#CD88137
Western diet	ssniff	Cat#TD88137

## Highlights

- CD epithelium exhibits increased transcriptional retinoid X receptor alpha (RXR**α**) activity
- **ω**−3 and **ω**−6 PUFAs in a Western diet induce transcriptional RXR**α** activity in IECs of mice
- PUFA-induced metabolic enteritis is mediated by RXR**α** in Paneth cells
- Isotretinoin ameliorates PUFA-induced enteritis and reduces the odds of developing CD

## References

[1] Dolinger M, Torres J, and Vermeire S (2024). Crohn’s disease. Lancet 403, 1177–1191. 10.1016/s0140-6736(23)02586-2. 38437854

[2] Adolph TE, Meyer M, Schwärzler J, Mayr L, Grabherr F, and Tilg H (2022). The metabolic nature of inflammatory bowel diseases. Nat. Rev. Gastroenterol. Hepatol 19, 753–767. 10.1038/s41575-022-00658-y. 35906289

[3] Roda G, Chien Ng S, Kotze PG, Argollo M, Panaccione R, Spinelli A, Kaser A, Peyrin-Biroulet L, and Danese S (2020). Crohn’s disease. Nat. Rev. Dis. Primers 6, 22. 10.1038/s41572-020-0156-2. 32242028

[4] Arifuzzaman M, Collins N, Guo CJ, and Artis D (2024). Nutritional regulation of microbiota-derived metabolites: Implications for immunity and inflammation. Immunity 57, 14–27. 10.1016/j.immuni.2023.12.009. 38198849 PMC10795735

[5] Muri J, and Kopf M (2021). Redox regulation of immunometabolism. Nat. Rev. Immunol 21, 363–381. 10.1038/s41577-020-00478-8. 33340021

[6] Sonnenburg JL, and Sonnenburg ED (2019). Vulnerability of the industrialized microbiota. Science 366, eaaw9255. 10.1126/science.aaw9255. 31649168

[7] Carter MM, Olm MR, Merrill BD, Dahan D, Tripathi S, Spencer SP, Yu FB, Jain S, Neff N, Jha AR, (2023). Ultra-deep sequencing of Hadza hunter-gatherers recovers vanishing gut microbes. Cell 186, 3111–3124.e13. 10.1016/j.cell.2023.05.046. 37348505 PMC10330870

[8] Braun T, Feng R, Amir A, Levhar N, Shacham H, Mao R, Hadar R, Toren I, Algavi Y, Abu-Saad K, (2024). Diet-omics in the Study of Urban and Rural Crohn disease Evolution (SOURCE) cohort. Nat. Commun 15, 3764. 10.1038/s41467-024-48106-6. 38704361 PMC11069498

[9] Jostins L, Ripke S, Weersma RK, Duerr RH, McGovern DP, Hui KY, Lee JC, Schumm LP, Sharma Y, Anderson CA, (2012). Host-microbe interactions have shaped the genetic architecture of inflammatory bowel disease. Nature 491, 119–124. 10.1038/nature11582. 23128233 PMC3491803

[10] de Lange KM, Moutsianas L, Lee JC, Lamb CA, Luo Y, Kennedy NA, Jostins L, Rice DL, Gutierrez-Achury J, Ji SG, (2017). Genome-wide association study implicates immune activation of multiple integrin genes in inflammatory bowel disease. Nat. Genet 49, 256–261. 10.1038/ng.3760. 28067908 PMC5289481

[11] Cordain L, Eaton SB, Sebastian A, Mann N, Lindeberg S, Watkins BA, O’Keefe JH, and Brand-Miller J (2005). Origins and evolution of the Western diet: health implications for the 21st century. Am. J. Clin. Nutr 81, 341–354. 10.1093/ajcn.81.2.341. 15699220

[12] Adolph TE, and Tilg H (2024). Western diets and chronic diseases. Nat. Med 30, 2133–2147. 10.1038/s41591-024-03165-6. 39085420

[13] Alagawany M, Elnesr SS, Farag MR, Abd El-Hack ME, Khafaga AF, Taha AE, Tiwari R, Yatoo MI, Bhatt P, Khurana SK, (2019). Omega-3 and omega-6 fatty acids in poultry nutrition: effect on production performance and health. Animals (Basel) 9, 573. 10.3390/ani9080573. 31426600 PMC6721126

[14] Zhao M, Feng R, Ben-Horin S, Zhuang X, Tian Z, Li X, Ma R, Mao R, Qiu Y, and Chen M (2022). Systematic review with meta-analysis: environmental and dietary differences of inflammatory bowel disease in Eastern and Western populations. Aliment. Pharmacol. Ther 55, 266–276. 10.1111/apt.16703. 34820868

[15] Schwärzler J, Mayr L, Grabherr F, Tilg H, and Adolph TE (2024). Epithelial metabolism as a rheostat for intestinal inflammation and malignancy. Trends Cell Biol. 34, 913–927. 10.1016/j.tcb.2024.01.004. 38341347

[16] Bevins CL, and Salzman NH (2011). Paneth cells, antimicrobial peptides and maintenance of intestinal homeostasis. Nat. Rev. Microbiol 9, 356–368. 10.1038/nrmicro2546. 21423246

[17] Adolph TE, Tomczak MF, Niederreiter L, Ko HJ, Böck J, Martinez-Naves E, Glickman JN, Tschurtschenthaler M, Hartwig J, Hosomi S, (2013). Paneth cells as a site of origin for intestinal inflammation. Nature 503, 272–276. 10.1038/nature12599. 24089213 PMC3862182

[18] Cadwell K, Liu JY, Brown SL, Miyoshi H, Loh J, Lennerz JK, Kishi C, Kc W, Carrero JA, Hunt S, (2008). A key role for autophagy and the autophagy gene Atg16l1 in mouse and human intestinal Paneth cells. Nature 456, 259–263. 10.1038/nature07416. 18849966 PMC2695978

[19] VanDussen KL, Liu TC, Li D, Towfic F, Modiano N, Winter R, Haritunians T, Taylor KD, Dhall D, Targan SR, (2014). Genetic variants synthesize to produce paneth cell phenotypes that define subtypes of Crohn’s disease. Gastroenterology 146, 200–209. 10.1053/j.gastro.2013.09.048. 24076061 PMC3899786

[20] Liu TC, Kern JT, Jain U, Sonnek NM, Xiong S, Simpson KF, VanDussen KL, Winkler ES, Haritunians T, Malique A, (2021). Western diet induces Paneth cell defects through microbiome alterations and farnesoid X receptor and type I interferon activation. Cell Host Microbe 29, 988–1001.e6. 10.1016/j.chom.2021.04.004. 34010595 PMC8192497

[21] Liang S, Guo XK, Ou J, Huang R, Xue Q, Zhang B, Chung Y, Wu W, Dong C, Yang X, (2019). Nutrient sensing by the intestinal epithelium orchestrates mucosal antimicrobial defense via translational control of Hes1. Cell Host Microbe 25, 706–718.e7. 10.1016/j.chom.2019.03.012. 31053533

[22] Jump DB, Tripathy S, and Depner CM (2013). Fatty acid-regulated transcription factors in the liver. Annu. Rev. Nutr 33, 249–269. 10.1146/annurev-nutr-071812-161139. 23528177 PMC3940310

[23] Lengqvist J, Mata De Urquiza A, Bergman AC, Willson TM, Sjövall J, Perlmann T, and Griffiths WJ (2004). Polyunsaturated fatty acids including docosahexaenoic and arachidonic acid bind to the retinoid X receptor alpha ligand-binding domain. Mol. Cell. Proteomics 3, 692–703. 10.1074/mcp.M400003-MCP200. 15073272

[24] Núñez V, Alameda D, Rico D, Mota R, Gonzalo P, Cedenilla M, Fischer T, Boscá L, Glass CK, Arroyo AG, (2010). Retinoid X receptor alpha controls innate inflammatory responses through the upregulation of chemokine expression. Proc. Natl. Acad. Sci. USA 107, 10626–10631. 10.1073/pnas.0913545107. 20498053 PMC2890831

[25] Altucci L, Leibowitz MD, Ogilvie KM, de Lera AR, and Gronemeyer H (2007). RAR and RXR modulation in cancer and metabolic disease. Nat. Rev. Drug Discov 6, 793–810. 10.1038/nrd2397. 17906642

[26] Evans RM, and Mangelsdorf DJ (2014). Nuclear receptors, RXR, and the Big Bang. Cell 157, 255–266. 10.1016/j.cell.2014.03.012. 24679540 PMC4029515

[27] de Urquiza AM, Liu S, Sjöberg M, Zetterström RH, Griffiths W, Sjövall J, and Perlmann T (2000). Docosahexaenoic acid, a ligand for the retinoid X receptor in mouse brain. Science 290, 2140–2144. 10.1126/science.290.5499.2140. 11118147

[28] Cao H, Li MY, Li G, Li SJ, Wen B, Lu Y, and Yu X (2020). Retinoid X receptor alpha regulates DHA-dependent spinogenesis and functional synapse formation in vivo. Cell Rep. 31, 107649. 10.1016/j.celrep.2020.107649. 32433958

[29] Repa JJ, Turley SD, Lobaccaro JA, Medina J, Li L, Lustig K, Shan B, Heyman RA, Dietschy JM, and Mangelsdorf DJ (2000). Regulation of absorption and ABC1-mediated efflux of cholesterol by RXR heterodimers. Science 289, 1524–1529. 10.1126/science.289.5484.1524. 10968783

[30] Ma F, Liu SY, Razani B, Arora N, Li B, Kagechika H, Tontonoz P, Núñez V, Ricote M, and Cheng G (2014). Retinoid X receptor α attenuates host antiviral response by suppressing type I interferon. Nat. Commun 5, 5494. 10.1038/ncomms6494. 25417649 PMC4380327

[31] Lukonin I, Serra D, Challet Meylan L, Volkmann K, Baaten J, Zhao R, Meeusen S, Colman K, Maurer F, Stadler MB, (2020). Phenotypic landscape of intestinal organoid regeneration. Nature 586, 275–280. 10.1038/s41586-020-2776-9. 33029001 PMC7116869

[32] Lloyd-Price J, Arze C, Ananthakrishnan AN, Schirmer M, Avila-Pacheco J, Poon TW, Andrews E, Ajami NJ, Bonham KS, Brislawn CJ, (2019). Multi-omics of the gut microbial ecosystem in inflammatory bowel diseases. Nature 569, 655–662. 10.1038/s41586-019-1237-9. 31142855 PMC6650278

[33] Mukherjee PK, Nguyen QT, Li J, Zhao S, Christensen SM, West GA, Chandra J, Gordon IO, Lin S, Wang J, (2023). Stricturing Crohn’s disease single-cell RNA sequencing reveals fibroblast heterogeneity and intercellular interactions. Gastroenterology 165, 1180–1196. 10.1053/j.gastro.2023.07.014. 37507073

[34] Layton A (2009). The use of isotretinoin in acne. Derm.Endocrinol. 1, 162–169. 10.4161/derm.1.3.9364. PMC2835909 20436884

[35] Müller-Dott S, Tsirvouli E, Vazquez M, Ramirez Flores RO, Badia-I-Mompel P, Fallegger R, Türei D, Lægreid A, and Saez-Rodriguez J (2023). Expanding the coverage of regulons from high-confidence prior knowledge for accurate estimation of transcription factor activities. Nucleic Acids Res. 51, 10934–10949. 10.1093/nar/gkad841. 37843125 PMC10639077

[36] Mayr L, Grabherr F, Schwärzler J, Reitmeier I, Sommer F, Gehmacher T, Niederreiter L, He GW, Ruder B, Kunz KTR, (2020). Dietary lipids fuel GPX4-restricted enteritis resembling Crohn’s disease. Nat. Commun 11, 1775. 10.1038/s41467-020-15646-6. 32286299 PMC7156516

[37] Schwärzler J, Mayr L, Vich Vila A, Grabherr F, Niederreiter L, Philipp M, Grander C, Meyer M, Jukic A, Tröger S, (2022). PUFA-induced metabolic enteritis as a fuel for Crohn’s disease. Gastroenterology 162, 1690–1704. 10.1053/j.gastro.2022.01.004. 35031299

[38] Harayama T, and Riezman H (2018). Understanding the diversity of membrane lipid composition. Nat. Rev. Mol. Cell Biol 19, 281–296. 10.1038/nrm.2017.138. 29410529

[39] Mathiowetz AJ, and Olzmann JA (2024). Lipid droplets and cellular lipid flux. Nat. Cell Biol 26, 331–345. 10.1038/s41556-024-01364-4. 38454048 PMC11228001

[40] Greupner T, Koch E, Kutzner L, Hahn A, Schebb NH, and Schuchardt JP (2019). Single-dose SDA-rich echium oil increases plasma EPA, DPAn3, and DHA concentrations. Nutrients 11, 2346. 10.3390/nu11102346. 31581725 PMC6835614

[41] Fu Y, Zhen J, and Lu Z (2017). Synergetic neuroprotective effect of docosahexaenoic acid and aspirin in SH-Y5Y by inhibiting miR-21 and activating RXRα and PPARα. DNA Cell Biol. 36, 482–489. 10.1089/dna.2017.3643. 28346830

[42] Khalil BD, Sanchez R, Rahman T, Rodriguez-Tirado C, Moritsch S, Martinez AR, Miles B, Farias E, Mezei M, Nobre AR, (2022). An NR2F1-specific agonist suppresses metastasis by inducing cancer cell dormancy. J. Exp. Med 219, e20210836. 10.1084/jem.20210836. 34812843 PMC8614154

[43] Loyo-Celis V, Patel D, Sanghvi S, Kaur K, Ponnalagu D, Zheng Y, Bindra S, Bhachu HR, Deschenes I, Gururaja Rao S, (2023). Biophysical characterization of chloride intracellular channel 6 (CLIC6). J. Biol. Chem 299, 105349. 10.1016/j.jbc.2023.105349. 37838179 PMC10641671

[44] Cunningham F, Allen JE, Allen J, Alvarez-Jarreta J, Amode MR, Armean IM, Austine-Orimoloye O, Azov AG, Barnes I, Bennett R, (2022). Ensembl 2022. Nucleic Acids Res. 50, D988–D995. 10.1093/nar/gkab1049. 34791404 PMC8728283

[45] Sandelin A, Alkema W, Engström P, Wasserman WW, and Lenhard B (2004). JASPAR: an open-access database for eukaryotic transcription factor binding profiles. Nucleic Acids Res. 32, D91–D94. 10.1093/nar/gkh012. 14681366 PMC308747

[46] Wang L, Zhang Y-L, Lin Q-Y, Liu Y, Guan X-M, Ma X-L, Cao H-J, Liu Y, Bai J, Xia Y-L, (2018). CXCL1–CXCR2 axis mediates angiotensin II-induced cardiac hypertrophy and remodelling through regulation of monocyte infiltration. Eur. Heart J 39, 1818–1831. 10.1093/eurheartj/ehy085. 29514257

[47] Ebisawa M, Umemiya H, Ohta K, Fukasawa H, Kawachi E, Christoffel G, Gronemeyer H, Tsuji M, Hashimoto Y, Shudo K, (1999). Retinoid X receptor-antagonistic diazepinylbenzoic acids. Chem. Pharm. Bull. (Tokyo) 47, 1778–1786. 10.1248/cpb.47.1778. 10748721

[48] Cesario RM, Klausing K, Razzaghi H, Crombie D, Rungta D, Heyman RA, and Lala DS (2001). The rexinoid LG100754 is a novel RXR:PPARgamma agonist and decreases glucose levels in vivo. Mol. Endocrinol 15, 1360–1369. 10.1210/mend.15.8.0677. 11463859

[49] Osburn DL, Shao G, Seidel HM, and Schulman IG (2001). Ligand-dependent degradation of retinoid X receptors does not require transcriptional activity or coactivator interactions. Mol. Cell. Biol 21, 4909–4918. 10.1128/mcb.21.15.4909-4918.2001. 11438648 PMC87210

[50] Goldstein JT, Dobrzyn A, Clagett-Dame M, Pike JW, and DeLuca HF (2003). Isolation and characterization of unsaturated fatty acids as natural ligands for the retinoid-X receptor. Arch. Biochem. Biophys 420, 185–193. 10.1016/j.abb.2003.09.034. 14622989

[51] Allenby G, Bocquel MT, Saunders M, Kazmer S, Speck J, Rosenberger M, Lovey A, Kastner P, Grippo JF, and Chambon P (1993). Retinoic acid receptors and retinoid X receptors: interactions with endogenous retinoic acids. Proc. Natl. Acad. Sci. USA 90, 30–34. 10.1073/pnas.90.1.30. 8380496 PMC45593

[52] Palchuk MB, London JW, Perez-Rey D, Drebert ZJ, Winer-Jones JP, Thompson CN, Esposito J, and Claerhout B (2023). A global federated real-world data and analytics platform for research. JAMIA Open 6, ooad035. 10.1093/jamiaopen/ooad035. 37193038 PMC10182857

[53] Paredes A, Justo-Méndez R, Jiménez-Blasco D, Núñez V, Calero I, Villalba-Orero M, Alegre-Martí A, Fischer T, Gradillas A, Sant’Anna VAR, (2023). gamma-Linolenic acid in maternal milk drives cardiac metabolic maturation. Nature 618, 365–373. 10.1038/s41586-023-06068-7. 37225978

[54] Duca FA, Waise TMZ, Peppler WT, and Lam TKT (2021). The metabolic impact of small intestinal nutrient sensing. Nat. Commun 12, 903. 10.1038/s41467-021-21235-y. 33568676 PMC7876101

[55] Feagan BG, Sandborn WJ, Mittmann U, Bar-Meir S, D’haens G, Bradette M, Cohen A, Dallaire C, Ponich TP, McDonald JWD, (2008). Omega-3 free fatty acids for the maintenance of remission in Crohn disease: the EPIC Randomized Controlled Trials. JAMA 299, 1690–1697. 10.1001/jama.299.14.1690. 18398081

[56] Crockett SD, Gulati A, Sandler RS, and Kappelman MD (2009). A causal association between isotretinoin and inflammatory bowel disease has yet to be established. Am. J. Gastroenterol 104, 2387–2393. 10.1038/ajg.2009.334. 19806085 PMC2775814

[57] Crockett SD, Porter CQ, Martin CF, Sandler RS, and Kappelman MD (2010). Isotretinoin use and the risk of inflammatory bowel disease: a case-control study. Am. J. Gastroenterol 105, 1986–1993. 10.1038/ajg.2010.124. 20354506 PMC3073620

[58] Shale M, Kaplan GG, Panaccione R, and Ghosh S (2009). Isotretinoin and intestinal inflammation: what gastroenterologists need to know. Gut 58, 737–741. 10.1136/gut.2008.170530. 19433589

[59] Ye X, Wu H, Sheng L, Liu YX, Ye F, Wang M, Zhou H, Su Y, and Zhang XK (2019). Oncogenic potential of truncated RXRalpha during colitis-associated colorectal tumorigenesis by promoting IL-6-STAT3 signaling. Nat. Commun 10, 1463. 10.1038/s41467-019-09375-8. 30931933 PMC6443775

[60] Desreumaux P, Dubuquoy L, Nutten S, Peuchmaur M, Englaro W, Schoonjans K, Derijard B, Desvergne B, Wahli W, Chambon P, (2001). Attenuation of colon inflammation through activators of the retinoid X receptor (RXR)/peroxisome proliferator-activated receptor gamma (PPARgamma) heterodimer. A basis for new therapeutic strategies. J. Exp. Med 193, 827–838. 10.1084/jem.193.7.827. 11283155 PMC2193371

[61] Keller H, Dreyer C, Medin J, Mahfoudi A, Ozato K, and Wahli W (1993). Fatty acids and retinoids control lipid metabolism through activation of peroxisome proliferator-activated receptor-retinoid X receptor heterodimers. Proc. Natl. Acad. Sci. USA 90, 2160–2164. 10.1073/pnas.90.6.2160. 8384714 PMC46045

[62] Chawla A, Repa JJ, Evans RM, and Mangelsdorf DJ (2001). Nuclear receptors and lipid physiology: opening the X-files. Science 294, 1866–1870. 10.1126/science.294.5548.1866. 11729302

[63] Hampe J, Franke A, Rosenstiel P, Till A, Teuber M, Huse K, Albrecht M, Mayr G, De La Vega FM, Briggs J, (2007). A genome-wide association scan of nonsynonymous SNPs identifies a susceptibility variant for Crohn disease in ATG16L1. Nat. Genet 39, 207–211. 10.1038/ng1954. 17200669

[64] Matsuzawa-Ishimoto Y, Yao X, Koide A, Ueberheide BM, Axelrad JE, Reis BS, Parsa R, Neil JA, Devlin JC, Rudensky E, (2022). The γδ IEL effector API5 masks genetic susceptibility to Paneth cell death. Nature 610, 547–554. 10.1038/s41586-022-05259-y. 36198790 PMC9720609

[65] Atreya R, and Siegmund B (2021). Location is important: differentiation between ileal and colonic Crohn’s disease. Nat. Rev. Gastroenterol. Hepatol 18, 544–558. 10.1038/s41575-021-00424-6. 33712743

[66] Ewels PA, Peltzer A, Fillinger S, Patel H, Alneberg J, Wilm A, Garcia MU, Di Tommaso P, and Nahnsen S (2020). The nf-core framework for community-curated bioinformatics pipelines. Nat. Biotechnol 38, 276–278. 10.1038/s41587-020-0439-x. 32055031

[67] Horn V, Cancino CA, Steinheuer LM, Obermayer B, Fritz K, Nguyen AL, Juhran KS, Plattner C, Bösel D, Oldenburg L, (2024). Multimodal profiling of peripheral blood identifies proliferating circulating effector CD4(+) T cells as predictors for response to integrin alpha4beta7-blocking therapy in inflammatory bowel disease. Gastroenterology 168, 327–343. 10.1053/j.gastro.2024.09.021. 39343250

[68] Hess MW, Pfaller K, Ebner HL, Beer B, Hekl D, and Seppi T (2010). 3D versus 2D cell culture implications for electron microscopy. Methods Cell Biol. 96, 649–670. 10.1016/s0091-679x(10)96027-5. 20869542

[69] Virshup I, Rybakov S, Theis FJ, Angerer P, and Wolf FA (2021). anndata: Annotated data. Preprint at bioRxiv. 10.1101/2021.12.16.473007.

[70] Virshup I, Bredikhin D, Heumos L, Palla G, Sturm G, Gayoso A, Kats I, Koutrouli M; Scverse Community, and Berger B, (2023). The scverse project provides a computational ecosystem for single-cell omics data analysis. Nat. Biotechnol 41, 604–606. 10.1038/s41587-023-01733-8. 37037904

[71] Sheng C, Lopes R, Li G, Schuierer S, Waldt A, Cuttat R, Dimitrieva S, Kauffmann A, Durand E, Galli GG, (2022). Probabilistic modeling of ambient noise in single-cell omics data. Preprint at bioRxiv. 10.1101/2022.01.14.476312.

[72] Bernstein NJ, Fong NL, Lam I, Roy MA, Hendrickson DG, and Kelley DR (2020). Solo: doublet identification in single-cell RNA-seq via semi-supervised deep learning. Cell Syst. 11, 95–101.e5. 10.1016/j.cels.2020.05.010. 32592658

[73] Gayoso A, Lopez R, Xing G, Boyeau P, Valiollah Pour Amiri V, Hong J, Wu K, Jayasuriya M, Mehlman E, Langevin M, (2022). A Python library for probabilistic analysis of single-cell omics data. Nat. Biotechnol 40, 163–166. 10.1038/s41587-021-01206-w. 35132262

[74] Wolf FA, Angerer P, and Theis FJ (2018). SCANPY: large-scale single-cell gene expression data analysis. Genome Biol. 19, 15. 10.1186/s13059-017-1382-0. 29409532 PMC5802054

[75] Xu C, Lopez R, Mehlman E, Regier J, Jordan MI, and Yosef N (2021). Probabilistic harmonization and annotation of single-cell transcriptomics data with deep generative models. Mol. Syst. Biol 17, e9620. 10.15252/msb.20209620. 33491336 PMC7829634

[76] Becht E, McInnes L, Healy J, Dutertre CA, Kwok IWH, Ng LG, Ginhoux F, and Newell EW (2018). Dimensionality reduction for visualizing single-cell data using UMAP. Nat. Biotechnol 37, 38–44. 10.1038/nbt.4314. 30531897

[77] Traag VA, Waltman L, and van Eck NJ (2019). From Louvain to Leiden: guaranteeing well-connected communities. Sci. Rep 9, 5233. 10.1038/s41598-019-41695-z. 30914743 PMC6435756

[78] Love MI, Huber W, and Anders S (2014). Moderated estimation of fold change and dispersion for RNA-seq data with DESeq2. Genome Biol. 15, 550. 10.1186/s13059-014-0550-8. 25516281 PMC4302049

[79] Badia-I-Mompel P, Vélez Santiago J, Braunger J, Geiss C, Dimitrov D, Müller-Dott S, Taus P, Dugourd A, Holland CH, Ramirez Flores RO, (2022). decoupleR: ensemble of computational methods to infer biological activities from omics data. Bioinform. Adv 2, vbac016. 10.1093/bioadv/vbac016. 36699385 PMC9710656

[80] Ignatiadis N, Klaus B, Zaugg JB, and Huber W (2016). Data-driven hypothesis weighting increases detection power in genome-scale multiple testing. Nat. Methods 13, 577–580. 10.1038/nmeth.3885. 27240256 PMC4930141

[81] Schubert M, Klinger B, Klünemann M, Sieber A, Uhlitz F, Sauer S, Garnett MJ, Blüthgen N, and Saez-Rodriguez J (2018). Perturbation-response genes reveal signaling footprints in cancer gene expression. Nat. Commun 9, 20. 10.1038/s41467-017-02391-6. 29295995 PMC5750219

[82] Dobin A, Davis CA, Schlesinger F, Drenkow J, Zaleski C, Jha S, Batut P, Chaisson M, and Gingeras TR (2013). STAR: ultrafast universal RNA-seq aligner. Bioinformatics 29, 15–21. 10.1093/bioinformatics/bts635. 23104886 PMC3530905

[83] Patro R, Duggal G, Love MI, Irizarry RA, and Kingsford C (2017). Salmon provides fast and bias-aware quantification of transcript expression. Nat. Methods 14, 417–419. 10.1038/nmeth.4197. 28263959 PMC5600148

[84] Manghi P, Blanco-Míguez A, Manara S, NabiNejad A, Cumbo F, Beghini F, Armanini F, Golzato D, Huang KD, Thomas AM, (2023). MetaPhlAn 4 profiling of unknown species-level genome bins improves the characterization of diet-associated microbiome changes in mice. Cell Rep. 42, 112464. 10.1016/j.celrep.2023.112464. 37141097 PMC10242440

[85] Beghini F, McIver LJ, Blanco-Míguez A, Dubois L, Asnicar F, Maharjan S, Mailyan A, Manghi P, Scholz M, Thomas AM, (2021). Integrating taxonomic, functional, and strain-level profiling of diverse microbial communities with bioBakery 3. eLife 10, e65088. 10.7554/eLife.65088. 33944776 PMC8096432

[86] Dixon P (2003). VEGAN, a package of R functions for community ecology. J. Veg. Sci 14, 927–930. 10.1111/j.1654-1103.2003.tb02228.x.

[87] Mallick H, Rahnavard A, McIver LJ, Ma S, Zhang Y, Nguyen LH, Tickle TL, Weingart G, Ren B, Schwager EH, (2021). Multivariable association discovery in population-scale meta-omics studies. PLOS Comput. Biol 17, e1009442. 10.1371/journal.pcbi.1009442. 34784344 PMC8714082

[88] Koeberle A, Shindou H, Harayama T, and Shimizu T (2010). Role of lysophosphatidic acid acyltransferase 3 for the supply of highly polyunsaturated fatty acids in TM4 Sertoli cells. FASEB J. 24, 4929–4938. 10.1096/fj.10-162818. 20705908

[89] Gollowitzer A, Pein H, Rao Z, Waltl L, Bereuter L, Loeser K, Meyer T, Jafari V, Witt F, Winkler R, (2025). Attenuated growth factor signaling during cell death initiation sensitizes membranes towards peroxidation. Nat. Commun 16, 1774. 10.1038/s41467-025-56711-2. 40000627 PMC11861335

[90] Koeberle A, Shindou H, Koeberle SC, Laufer SA, Shimizu T, and Werz O (2013). Arachidonoyl-phosphatidylcholine oscillates during the cell cycle and counteracts proliferation by suppressing Akt membrane binding. Proc. Natl. Acad. Sci. USA 110, 2546–2551. 10.1073/pnas.1216182110. 23359699 PMC3574958

[91] Koeberle A, Pergola C, Shindou H, Koeberle SC, Shimizu T, Laufer SA, and Werz O (2015). Role of p38 mitogen-activated protein kinase in linking stearoyl-CoA desaturase-1 activity with endoplasmic reticulum homeostasis. FASEB J. 29, 2439–2449. 10.1096/fj.14-268474. 25678624

[92] Thürmer M, Gollowitzer A, Pein H, Neukirch K, Gelmez E, Waltl L, Wielsch N, Winkler R, Löser K, Grander J, (2022). PI(18:1/18:1) is a SCD1-derived lipokine that limits stress signaling. Nat. Commun 13, 2982. 10.1038/s41467-022-30374-9. 35624087 PMC9142606

[93] Koeberle SC, Thürmer M, Su F, Werner M, Grander J, Hofer L, Gollowitzer A, Xuan LL, Benscheid FJ, Bonyadi Rad E, (2025). Silybin A from Silybum marianum reprograms lipid metabolism to induce a cell fate-dependent class switch from triglycerides to phospholipids. Theranostics 15, 2006–2034. 10.7150/thno.99562. 39897559 PMC11780512

