# Supplementary material for: Metabolic stress sensing by epithelial RXRα links westernization of diet with Crohn’s disease
Source: Cell Metab. Author manuscript; Available in PMC 2026 Mar 30. (PMC13034575; doi:10.1016/j.cmet.2025.11.008)
Supplement: Document S1. Figures S1–S8, Tables S5–S7, and TRR241 IBDome Consortium members [file NIHMS2140806-supplement-Document_S1__Figures_S1_S8__Tables_S5_S7__and_TRR241_IBDome_Consortium_members.pdf]

Cell Metabolism, Volume *38*

Supplemental information

Metabolic stress sensing by epithelial RXRα links westernization of diet with Crohn's disease

Moritz Meyer, Felix Grabherr, Christina Plattner, Michel V. Hadjihannas, Zhigang Rao, Valentin Marteau, Víctor Alonso López-Agudelo, Julian Schwärzler, Lisa Mayr, Almina Jukic, Laura Scheffauer, Luis Zundel, Barbara Enrich, Alexandra Pfister, Anna Simonini, Christoph Grander, Richard Hilbe, David Haschka, Andreas Zollner, Kathrin Vouk, Patrizia Moser, Michael W. Hess, Niloofar Nemati, Dietmar Rieder, Felix Sommer, Philip Rosenstiel, Qitao Ran, Richard S. Blumberg, Arthur Kaser, Florian Rieder, Andreas Koeberle, Christoph Becker, Raja Atreya, Anja A. Kühl, Britta Siegmund, Andre Franke, The IBDome Consortium, Herbert Tilg, Zlatko Trajanoski, and Timon E. Adolph

Supplemental Information

Supplemental Figure Titles:

Figure S1. Transcriptional profiling of small intestinal CD. Related to Figure 1.

Figure S2. Expression of *Rxrα* and related genes in our model. Related to Figure 2.

Figure S3. Dietary PUFAs accumulate in epithelial scrapings of *Gpx4*$^{+/-IEC}$ mice exposed to a PUFA-enriched Western diet and dose-dependently exhibit close proximity to RXRα. Related to Figure 3.

Figure S4. *Gpx4*-deficiency in Paneth cells does not affect their exocrine function. Related to Figure 4.

Figure S5. Shotgun sequencing of small intestinal content after 4-week exposure to a PUFA-enriched Western diet. Related to Figure 4.

Figure S6. Intestinal epithelial RXRα drives CXCL1 production independent from lipid peroxidation and cellular stress signaling. Related to Figure 5.

Figure S7. *Gpx4*-deficient macrophages and dendritic cells did not express pro-inflammatory cytokines upon PUFA exposure. Related to Figure 5.

Figure S8. Intestinal epithelial *Rxrα*-deficiency did not affect susceptibility to experimental colitis. Related to Figure 5.

Data S1. Unprocessed data underlying the display items in the manuscript, related to Figures 1-6 and S1-S8**.**

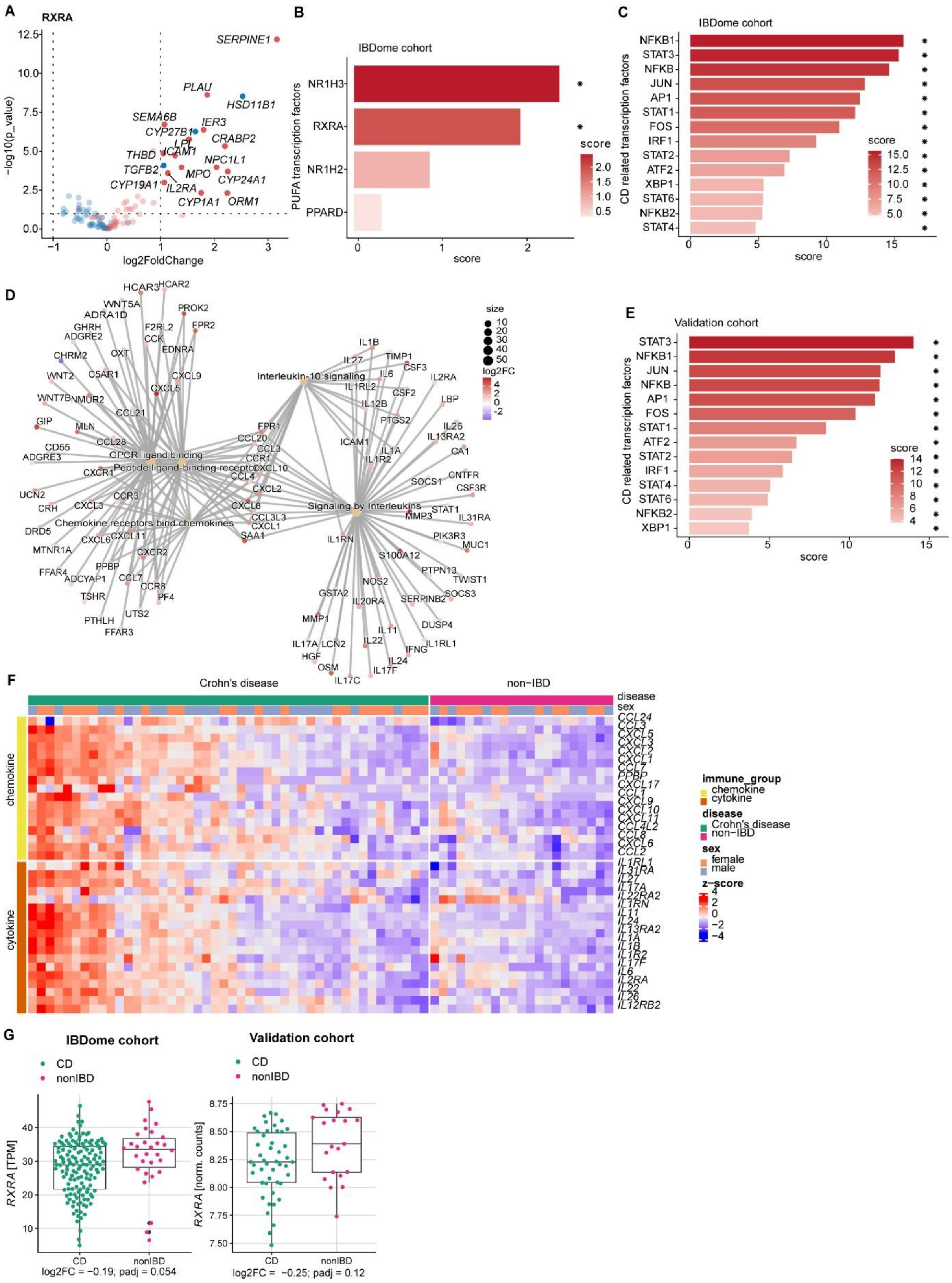

Figure S1. Transcriptional profiling of small intestinal CD. Related to Figure 1.

(A) Volcano plot showing the expression of RXRα target genes (color indicates the mode of regulation: inhibition = blue, activation = red) in the validation cohort [1] in CD patients compared to non-IBD controls.

(B) Transcription factor activities of long-chain fatty acid sensing transcription factors inferred by CollecTRI in the small intestinal mucosa of overweight/obese patients (BMI ≥25) as compared to non-overweight/non-obese patients (BMI <25) in the 'IBDome' discovery cohort.

(C) Transcription factor activities of CD-related transcription factors in the small intestinal mucosa of CD patients compared to non-IBD controls in the 'IBDome' discovery cohort.

(D) cnetplot depicting significant functional enrichment results (Reactome database) related to chemokine and interleukin signaling in the small intestinal mucosa of CD patients compared to non IBD controls in the 'IBDome' discovery cohort.

(E) CD-related transcription factor activities in the small intestinal mucosa of CD patients compared to non-IBD controls in the validation cohort [1].

(F) Heatmap depicting differentially expressed cytokines and chemokines in the small intestinal mucosa of CD patients compared to non-IBD controls in the validation cohort [1].

(G) *RXRA* expression in CD patients compared to non-IBD controls in the 'IBDome' discovery cohort and the validation cohort [1].

*FDR<0.1.

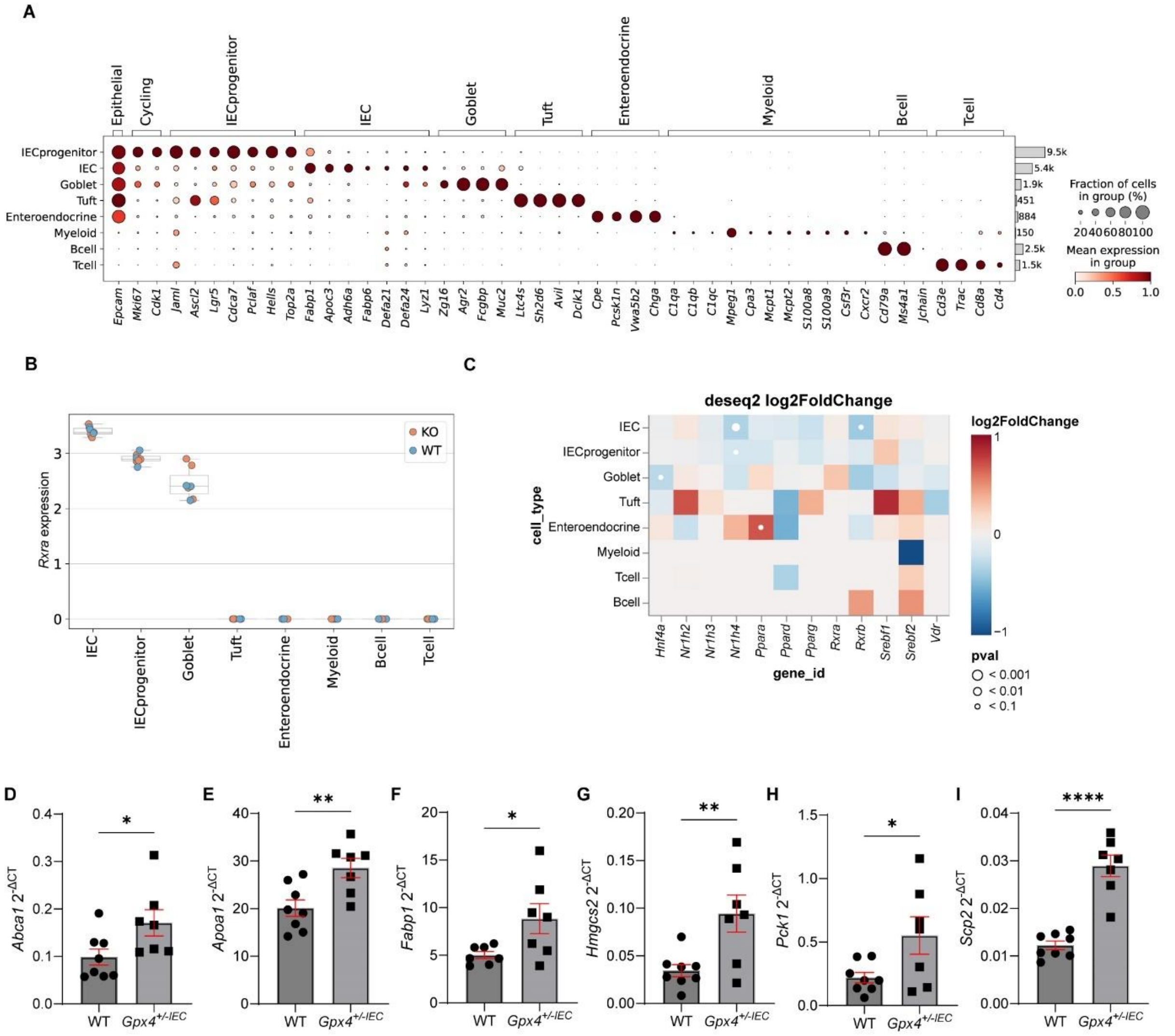

Figure S2. Expression of *Rxrα* and related genes in our model. Related to Figure 2.

(A) Dot plot showing relative gene-expression of selected cell-type specific markers, size of the dot indicates percentage of fraction of cells, mean expression is color coded.

(B) *Rxra* expression in different cell types of *Gpx4*$^{+/-IEC}$ and WT mice (WT n=3, *Gpx4*$^{+/-IEC}$ n=4). Each dot represents the mean of a sample.

(C) Heatmap of indicated gene expression in different cell types of *Gpx4*$^{+/-IEC}$ as compared to WT mice determined by single-cell RNA sequencing (WT n=3, *Gpx4*$^{+/-IEC}$ n=4). Red color indicates upregulation compared to WT mice. The white circle indicates statistical significance. Note that *Rxra* is not differentially expressed in IECs from *Gpx4*$^{+/-IEC}$ mice.

(D-I) Expression of *Abca1* (D), *Apoa1* (E), *Fabp1* (F), *Hmgcs2* (G), *Pck1* (H) and *Scp2* (I) in small intestinal scrapings of *Gpx4*$^{+/-IEC}$ compared to WT mice (n≥7 in each group) exposed to a PUFA-enriched Western diet for 3 months as determined by qPCR.

(D, E, F, G, H, I) Data represented as mean ± SEM (unpaired Student's t-test).

**P*<0.05; ***P*<0.01; *****P*<0.0001.

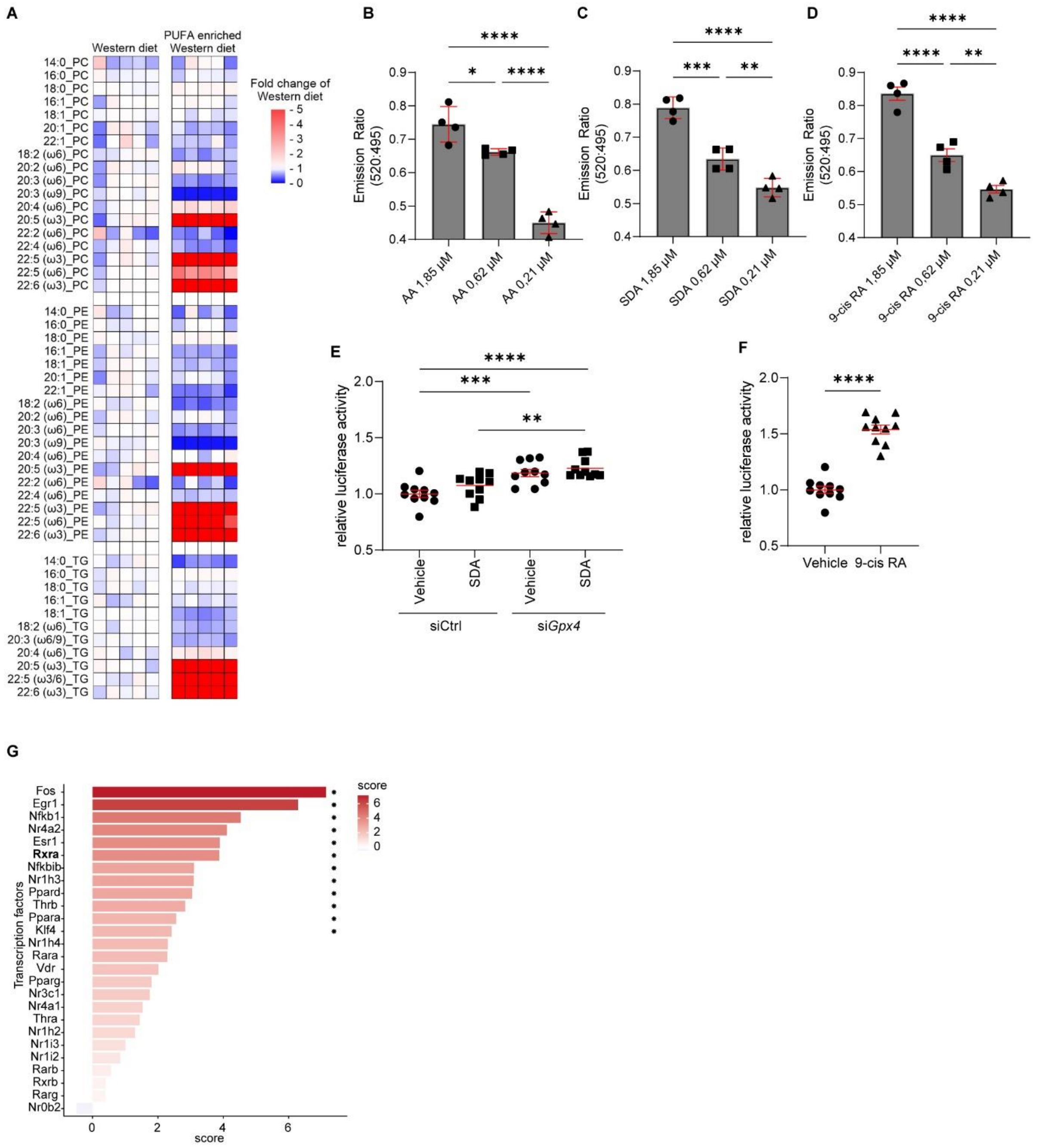

Figure S3. Dietary PUFAs accumulate in epithelial scrapings of *Gpx4*$^{+/-IEC}$ mice exposed to a PUFA-enriched Western diet and dose-dependently exhibit close proximity to RXRα. Related to Figure 3.

(A) Heatmap depicting changes in the proportion of fatty acids within PC, PE and TG species in small intestinal scrapings from *Gpx4*$^{+/-IEC}$ mice fed a PUFA-enriched Western diet or a Western diet without PUFA enrichment for 3 months (n=5).

(B-D) TR-FRET assay depicting a concentration-dependent proximity of AA (B), SDA (C) and 9-cis retinoic acid (D) with RXRα (n=4).

(E) Relative RXRα transcription factor activity, as assessed by a lentiviral luciferase reporter, in si*Gpx4* and siCtrl MODE-K IECs after stimulation with vehicle or steardionic acid for 24h. Note that the vehicle group is the same as in Figure 3G (n=10).

(F) Relative RXRα transcription factor activity, as assessed by a lentiviral luciferase reporter, in siCtrl MODE-K IECs after stimulation with vehicle or 9-cis retinoic acid for 24h. Note that vehicle group is the same as in Figure 3G (n=10).

(G) Estimated transcription factor activity of docosahexaenoic acid-stimulated si*Gpx4* MODE-K IECs for 8h, compared to siCtrl IECs as assessed by applying a univariate linear model using CollecTRI after bulk RNA sequencing (n= 6; 3 si*Gpx4* vs 3 siCtrl, Benjamini-Hochberg *P-adjusted<0.1).

(A) Individual data are shown.

(B, C, D, E) Data represented as mean ± SEM (one-way ANOVA with post-hoc Bonferroni).

(F) Data represented as mean ± SEM (unpaired Student's t-test).

**P*<0.05 or FDR<0.1; ***P*<0.01; ****P*<0.001; *****P*<0.0001.

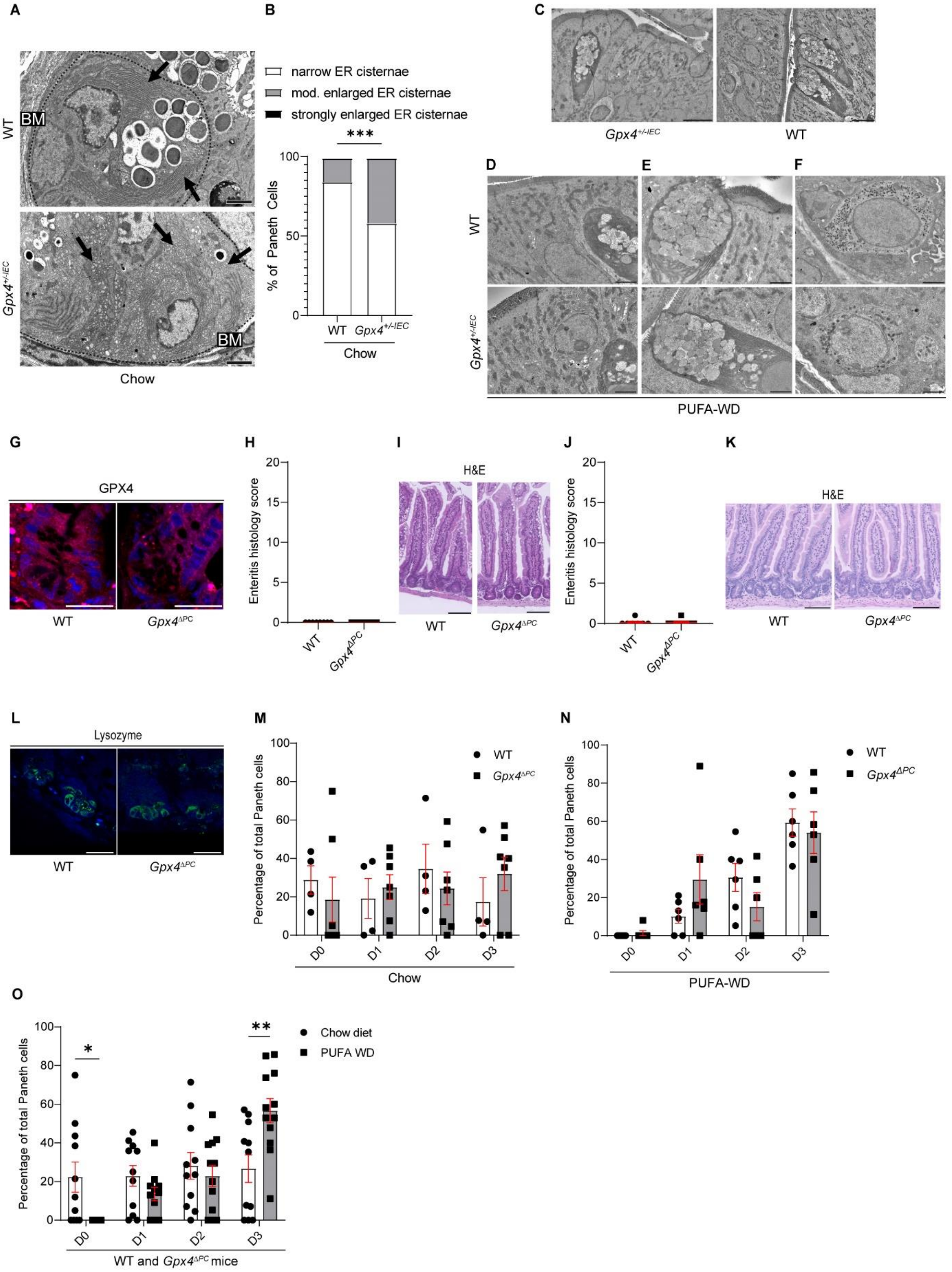

Figure S4. *Gpx4*-deficiency in Paneth cells does not affect their exocrine function. Related to Figure 4.

(A and B) Representative transmission electron microscopy images of Paneth cells (marked by dotted line) from *Gpx4*$^{+/-IEC}$ and WT control mice after 3 months of Chow diet feeding (A) with quantification of

endoplasmic reticulum dilatation of Paneth cells in (B). Scale bars indicate 2 μm; BM indicates the basement membrane (n=3 mice per genotype).

(C-F) Representative transmission electron microscopy images of intestinal epithelial cells: low magnification overview (C) with enterocytes (D), goblet cells (E) and enteroendocrine cells (F) from WT and *Gpx4*$^{+/-IEC}$ mice after a 3 months exposure to a PUFA-enriched Western diet demonstrating no ultrastructural alterations. Scale bars indicate 5μm in (C) and 2μm in (D-F).

(G) GPX4 immunolabeling of crypt IECs from WT and *Gpx4*$^{\Delta PC}$ mice confirming GPX4 knockout. Scale bars indicate 25 μm.

(H-K) Enteritis histology score and representative H&E images of the small intestine of WT and *Gpx4*$^{\Delta PC}$ mice fed a Western-diet (H and I) (WT n=8 , *Gpx4*$^{\Delta PC}$ n=10) or a low-fat diet (J and K) (WT n=7, *Gpx4*$^{\Delta PC}$ n=7); each dot represents an experimental animal. Scale bars indicate 100 μm.

(L) Representative images of lysozyme immunolabelled Paneth cells from WT and *Gpx4*$^{\Delta PC}$ mice fed a Chow diet. Scale bars indicate 50 μm.

(M, N) Percentage of Paneth cells with the indicated pattern of lysozyme staining in WT and *Gpx4*$^{\Delta PC}$ mice fed a chow diet (M) (WT n=4, *Gpx4*$^{\Delta PC}$ n=7) or a PUFA-WD (N) (WT n=6, *Gpx4*$^{\Delta PC}$ n=6). D0 indicates a normal lysozyme pattern, D1 indicates a disordered lysozyme expression, D2 indicates a depleted lysozyme expression and D3 indicates a diffuse lysozyme expression as previously defined [2].

(O) Percentage of Paneth cells with the indicated pattern of lysozyme staining in both, WT and *Gpx4*$^{\Delta PC}$ mice fed a Chow diet as compared to a PUFA-enriched Western diet (n≥11 in each group).

(B) Data represented as mean ± SEM (unpaired Student's t-test).

(H, J) Data represented as median (Mann-Whitney U test).

(M, N, O) Data represented as mean ± SEM (one-way ANOVA with post-hoc Bonferroni).

*P<0.05; **P<0.01; ***P<0.001.

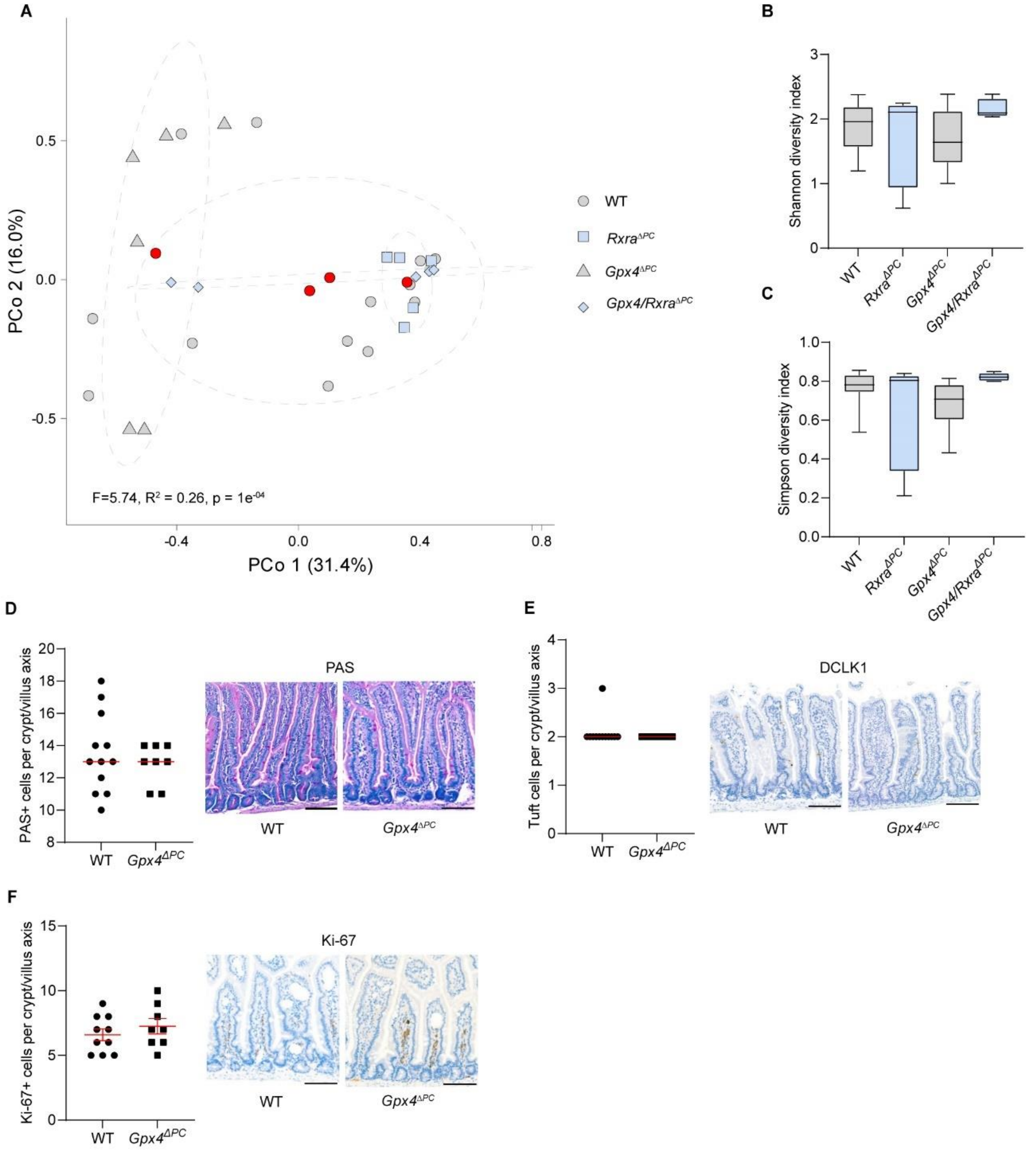

Figure S5. Shotgun sequencing of small intestinal content after 4-week exposure to a PUFA-enriched Western diet. Related to Figure 4.

(A) Microbiome β-diversity visualized by principal coordinate analysis of WT, *Rxra^{ΔPC}, Gpx4^{ΔPC}* and *Gpx4/Rxra^{ΔPC}* mice after 4-week exposure to a PUFA-enriched Western diet (n=14/5/6/5). Red dots represent the centroids of the groups. We did not note significant differences between genotypes tested with PERMANOVA (Adonis 2) with 10000 permutations.

(B and C) Shannon (B) and Simpson (C) diversity indices of small intestinal microbiota samples of WT, *Rxra^{ΔPC}, Gpx4^{ΔPC}* and *Gpx4/Rxra^{ΔPC}* mice fed a PUFA-enriched Western diet for 4 weeks indicating no significant difference in microbial alpha-diversity between the genotypes. Note that WT and *Gpx4^{ΔPC}* mice are also shown in Figure 4K (n=14/5/6/5).

(D-F) Numbers of PAS^{+} goblet cells (D) (WT n=12, *Gpx4^{ΔPC}* n=8), DCLK1^{+} tuft cells (E) (WT n=12, *Gpx4^{ΔPC}* n=8), or the amount of proliferating IECs (labelled by Ki67) (F) (WT n=10, *Gpx4^{ΔPC}* n=8) per crypt villus axis in *Gpx4^{ΔPC}* and WT mice exposed to a PUFA-enriched Western diet and representative images of the immunolabelling. Scale bars indicate 100 µm.

(B, C) Data represented as mean ± SEM (one-way ANOVA with post-hoc Bonferroni).

(D, E) Data represented as median (Mann-Whitney U test).

(F) Data represented as mean ± SEM (unpaired Student's t-test).

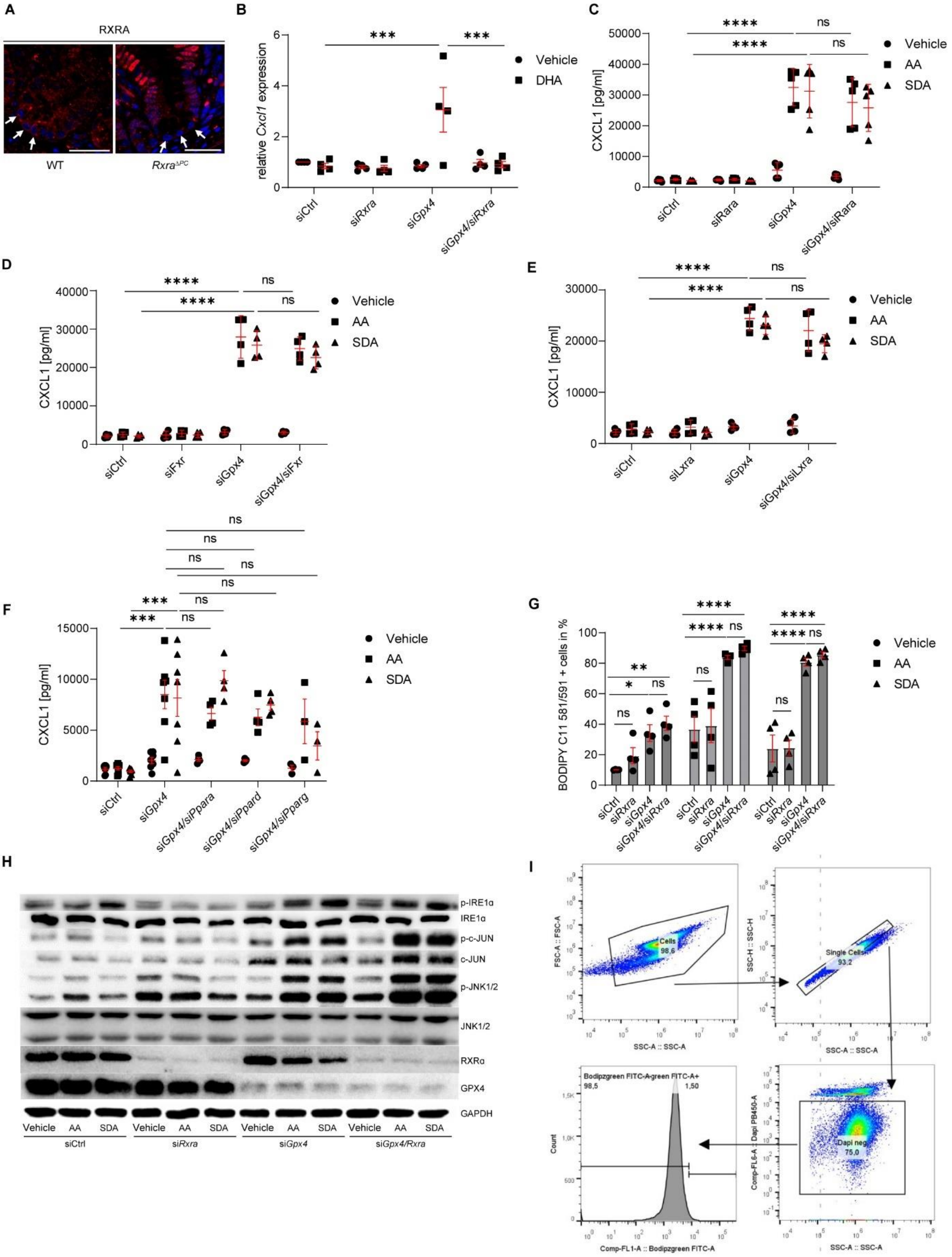

Figure S6. Intestinal epithelial RXRα drives CXCL1 production independent from lipid peroxidation and cellular stress signaling. Related to Figure 5.

(A) RXRA immunolabelling of WT and *Rxra*$^{\Delta PC}$ mice showing *Rxra* knockout specifically in crypt IECs. Scale bars indicate 25 µm.

(B) *Cxcl1* expression of indicated MODE-K IECs stimulated with vehicle or DHA and determined by qPCR after 8 hours of stimulation (n=4).

(C-F) CXCL1 quantification in the supernatant of PUFA-stimulated IECs with co-silencing of interacting transcription factors: si*Rara* (B)*,* si*Fxr* (C) si*Lxra* (D) si*Ppara*, si*Ppard* and si*Pparg* (E) after 24 hours as assessed with ELISA (B n=5, C and D n=4, E n≥3).

(G) Lipid peroxidation quantification in of indicated IECs stimulated with vehicle, AA and SDA for 24h, as assessed with BODIPYC11 581/591 labelling (n≥4).

(H) Immunoblot depicting endoplasmic reticulum stress and MAPK activity in indicated IECs after PUFA exposure for 24h (n≥3 independent experiments).

(I) Flow cytometry gating strategy for single, DAPI^{-} and BODIPYC11 581/591^{+} MODE-K IECs.

(B, C, D, E, F, G) Data represented as mean ± SEM (one-way ANOVA with post-hoc Bonferroni).

ns not significant; **P*<0.05; ***P*<0.01; ****P*<0.001; *****P*<0.0001

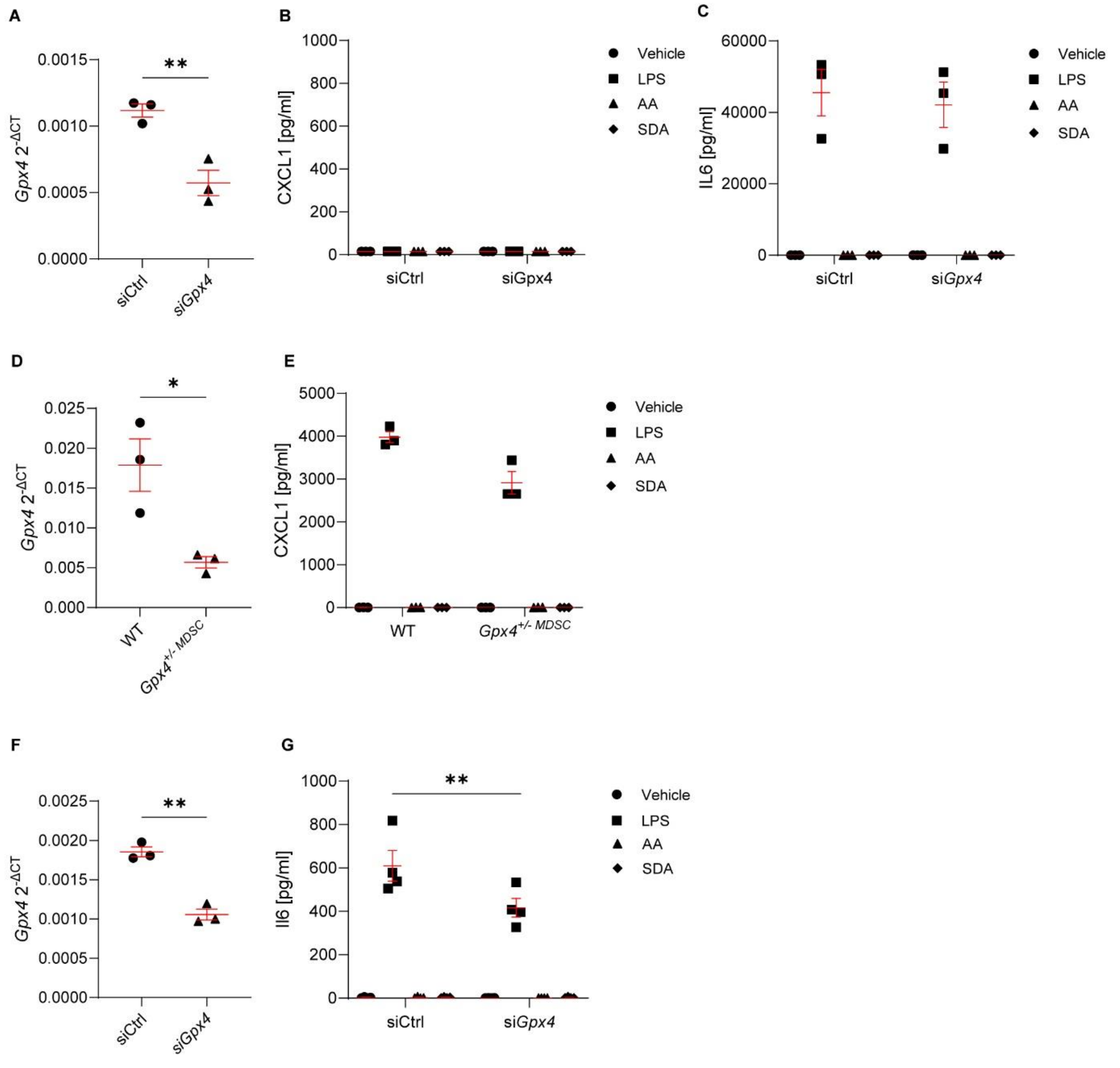

Figure S7. *Gpx4*-deficient macrophages and dendritic cells did not express pro-inflammatory cytokines upon PUFA exposure. Related to Figure 5.

(A) *Gpx4* expression of siCtrl and si*Gpx4* RAW 264.7 macrophages (n=3) as determined by qPCR.

(B) CXCL1 quantification of indicated siCtrl and si*Gpx4* RAW 264.7 macrophages (n=3) stimulated with vehicle, LPS, AA or SDA for 24h, as determined in the supernatant by ELISA.

(C) IL6 quantification of indicated siCtrl and si*Gpx4* RAW 264.7 macrophages (n=3) stimulated with vehicle, LPS, AA or SDA for 24h, as determined in the supernatant by ELISA.

(D) *Gpx4* expression of BMDMs of WT and $Gpx4^{+/- MDSC}$ mice (n=3) as determined by qPCR.

(E) CXCL1 quantification of indicated BMDMs of WT and $Gpx4^{+/- MDSC}$ mice (n=3) stimulated with vehicle, LPS, AA or SDA for 24h, as determined in the supernatant by ELISA.

(F) *Gpx4* expression of siCtrl and si*Gpx4* DC2.4 dendritic cells (n=3) as determined by qPCR.

(G) IL6 quantification of indicated siCtrl and si*Gpx4* DC2.4 dendritic cells (n=4) stimulated with vehicle, LPS, AA or SDA for 24h, as determined in the supernatant by ELISA.

(A, D, F) Data represented as mean ± SEM (unpaired Student's t-test).

(B, C, E, G) Data represented as mean ± SEM (one-way ANOVA with post-hoc Bonferroni).

*P<0.05; **P<0.01

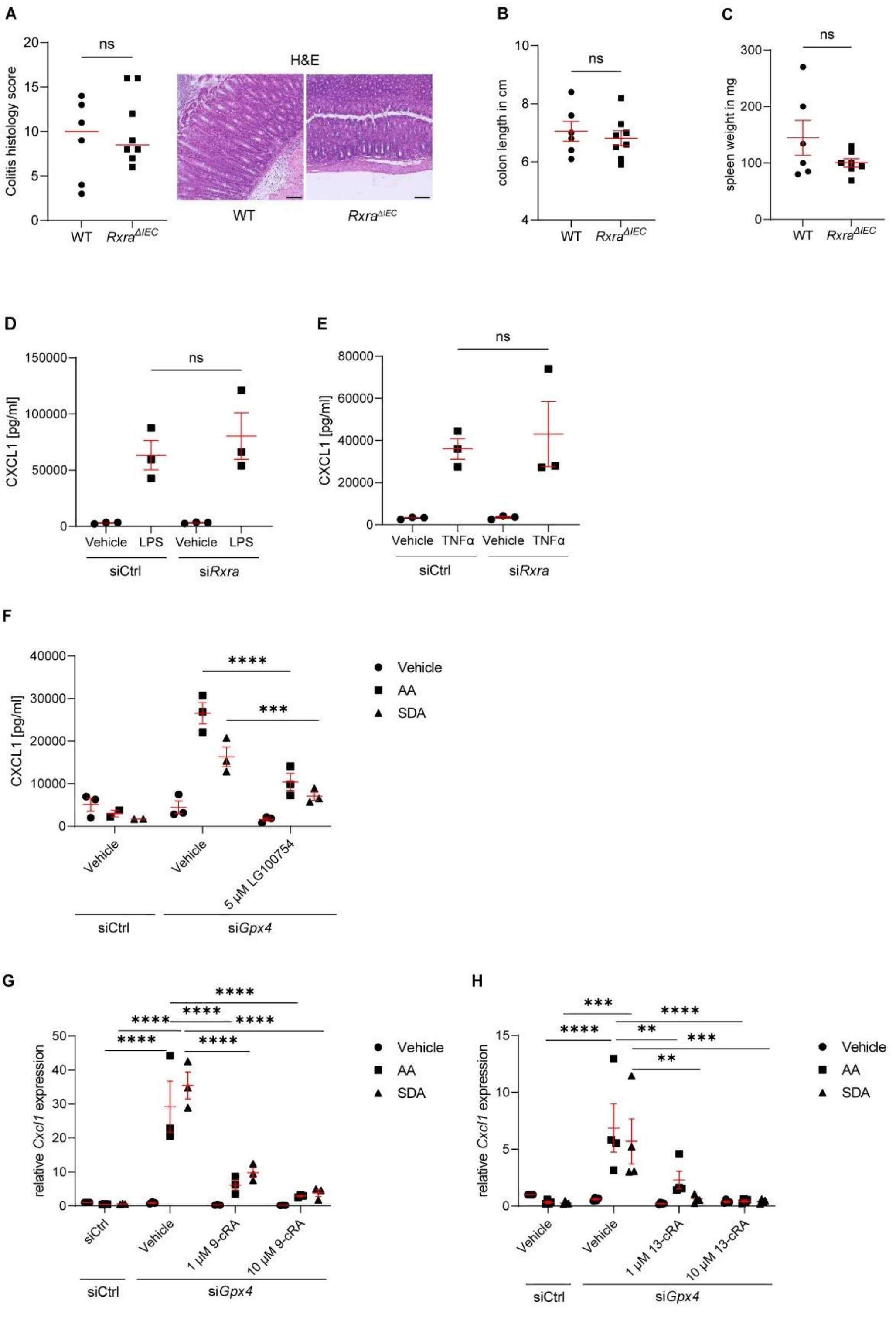

Figure S8. Intestinal epithelial *Rxrα*-deficiency did not affect susceptibility to experimental colitis. Related to Figure 5.

(A) Colitis histology score and representative H&E images of WT and *Rxra*$^{\Delta IEC}$ mice after DSS colitis induction. Scale bars indicate 100 μm (WT n=6, *Rxra*$^{\Delta IEC}$ n=8).

(B and C) Colon length (B) and spleen weight (C) of WT and *Rxra*$^{\Delta IEC}$ mice after DSS colitis induction (B, WT n=6, *Rxra*$^{\Delta IEC}$ n=8; C, WT n=6, *Rxra*$^{\Delta IEC}$ n=7).

(D and E) CXCL1 quantification of siCtrl*,* and si*Rxra* MODE-K IECs stimulated with LPS (D) and TNF (E) for 24h, as determined in the supernatant by ELISA (n=3). Note that the vehicle group is the same in (D) and (E).

(F) CXCL1 quantification of indicated MODE-K IECs stimulated with vehicle, AA or SDA and LG100754 for 24h, as determined in the supernatant by ELISA (n=3).

(G and H) *Cxcl1* expression of indicated MODE-K IECs stimulated with vehicle, AA or SDA and 9-cis retinoic acid or vehicle (G, n=3) or 13-cis retinoic acid or vehicle (H, n=4) for 24h, as determined by qPCR.

(A) Data represented as median (Mann-Whitney U test).

(B, C, D, E) Data represented as mean ± SEM (unpaired Student’s t-test).

(F, G, H) Data represented as mean ± SEM (one-way ANOVA with post-hoc Bonferroni).

ns, not significant; ***P*<0.01; ****P*<0.001; *****P*<0.0001

Table S5. Relative abundance of polyunsaturated fatty acids (PUFA) in the Western diet (WD) and the PUFA-enriched Western Diet supplemented with 10% fish oil (PUFA-WD). Related to STAR Methods.

PUFA	Western diet (%)	PUFA-enriched Western diet (%)
C18:2 (ω6)	0,38	0,41
C18:3 (ω3)	0,11	0,15
C18:4 (ω3)	-	0,30
C20:4 (ω6)	-	0,09
C20:5 (ω3)	-	1,75
C22:5 (ω3)	-	0,19
C22:6 (ω3)	-	1,21

Table S6. Primers. Related to STAR Methods.

Mouse	Forward	Reverse
beta-actin	GATGCTCCCCGGGCTGTATT	GGGGTACTTCAGGGTCAGGA
Cxcl1	CTGGGATTCACCTCAAGAACATC	CAGGGTCAAGGCAAGCCTC
Gpx4	GATGGAGCCCATTCCTGAACC	CCCTGTACTTATCCAGGCAGA
Hmgcs2	TTGCCCTGGAGGTCTATTTTCC	GAAGCCCATACGGGTCTGG
Apoa1	GGCACGTATGGCAGCAAGAT	CCAAGGAGGAGGATTCAAACTG
Pck1	CTGCATAACGGTCTGGACTTC	CAGCAACTGCCCGTACTCC
Scp2	CCTTCTGTCGCTTTGAAATCTCC	GCTTCCTTTGCCATATCAGGAT
Abca1	GCTTGTTGGCCTCAGTTAAGG	GTAGCTCAGGCGTACAGAGAT
Fabp1	AAGGCAGTCGTCAAGCTGG	CATTGAGTTCAGTCACGGACTT

Table S7. Mass spectrometry parameters of the QTRAP6500+ system. Related to STAR Methods.

**Parameters**	**PC**	**PE**	**TG**
Curtain gas (CUR)	40 psi	40 psi	40 psi
Collision gas (CAD)	medium	medium	medium
Ion spray voltage (IS)	-4,500 V	-4,500 V	5,500 V
Heated capillary temperature (TEM)	350°C	650°C	400°C
Sheath gas pressure (GS1)	55 psi	55 psi	60 psi
Auxiliary gas (GS2)	75 psi	75 psi	70 psi
Declustering potential (DP)	-44 V	-50 V	120 V
Entrance potential (EP)	-10 V	-10 V	10 V
Collision energy (CE)	-46 eV	-38 eV	35 eV
Collision cell exit potential (CXP)	-11 V	-12 V	26 V

TRR241 IBDome Consortium members

Imke Atreya[1], Petra Bacher[2,3], Christian Bojarski[4], Nathalie Britzen-Laurent[1], Caroline Bosch-Voskens[1], Hyun-Dong Chang[5], Andreas Diefenbach[6], Claudia Günther[1], Ahmed N. Hegazy[4], Kai Hildner[1], Christoph S. N. Klose[6], Kristina Koop[1], Susanne Krug[4], Moritz Leppkes[1], Rocío López-Posadas[1], Leif S.-H. Ludwig[7], Clemens Neufert[1], Markus Neurath[1], Jay V. Patankar[1], Magdalena Prüß[3], Andreas Radbruch[5], Chiara Romagnani[3], Francesca Ronchi[6], Ashley D. Sanders[4,8], Alexander Scheffold[2], Jörg-Dieter Schulzke[4], Michael Schumann[4], Sebastian Schürmann[1], Michael Stürzl[1], Antigoni Triantafyllopoulou[5,9], Maximilian Waldner[1], Carl Weidinger[4], Stefan Wirtz[1], Sebastian Zundler[1]

[1]Department of Medicine 1, Friedrich-Alexander University, Erlangen, Germany

[2]Institute of Clinical Molecular Biology, Christian-Albrecht University of Kiel, Kiel, Germany.

[3]Institute of Immunology, Christian-Albrecht University of Kiel and UKSH Schleswig-Holstein, Kiel, Germany.

[4]Charité – Universitätsmedizin Berlin, Freie Universität Berlin and Humboldt-Universität zu Berlin, Department of Gastroenterology, Infectious Diseases and Rheumatology, Berlin, Germany

[5]Deutsches Rheuma-Forschungszentrum, ein Institut der Leibniz-Gemeinschaft, Berlin, Germany

[6]Charité – Universitätsmedizin Berlin, Freie Universität Berlin and Humboldt-Universität zu Berlin, Institute of Microbiology, Infectious Diseases and Immunology

[7]Berlin Institute für Gesundheitsforschung, Medizinische System Biologie, Charité – Universitätsmedizin Berlin

[8]Max Delbrück Center für Molekulare Medizin, Charité – Universitätsmedizin Berlin

[9]Charité – Universitätsmedizin Berlin, Freie Universität Berlin and Humboldt-Universität zu Berlin, Department of Rheumatology and Clinical Immunology, Berlin, Germany

Supplemental References

[1] Lloyd-Price, J., Arze, C., Ananthakrishnan, A.N., Schirmer, M., Avila-Pacheco, J., Poon, T.W., Andrews, E., Ajami, N.J., Bonham, K.S., Brislawn, C.J., et al. (2019). Multi-omics of the gut microbial ecosystem in inflammatory bowel diseases. Nature *569*, 655-662. 10.1038/s41586-019-1237-9.

[2] Cadwell, K., Liu, J.Y., Brown, S.L., Miyoshi, H., Loh, J., Lennerz, J.K., Kishi, C., Kc, W., Carrero, J.A., Hunt, S., et al. (2008). A key role for autophagy and the autophagy gene Atg16l1 in mouse and human intestinal Paneth cells. Nature *456*, 259-263. 10.1038/nature07416.
