# Supplementary material for: Metabolic stress sensing by epithelial RXRα links westernization of diet with Crohn’s disease
Source: Cell Metab. Author manuscript; Available in PMC 2026 Mar 30. (PMC13034575; doi:10.1016/j.cmet.2025.11.008)
Supplement: Data S1. Unprocessed data underlying the display items in the manuscript, related to Figures 1–6 and S1–S8 [file NIHMS2140806-supplement-Data_S1__Unprocessed_data_underlying_the_display_items_in_the_manuscript__related_to_Figures_1_6_and_S1_S8.zip › Uncropped Blots.pdf]

Figure S6H

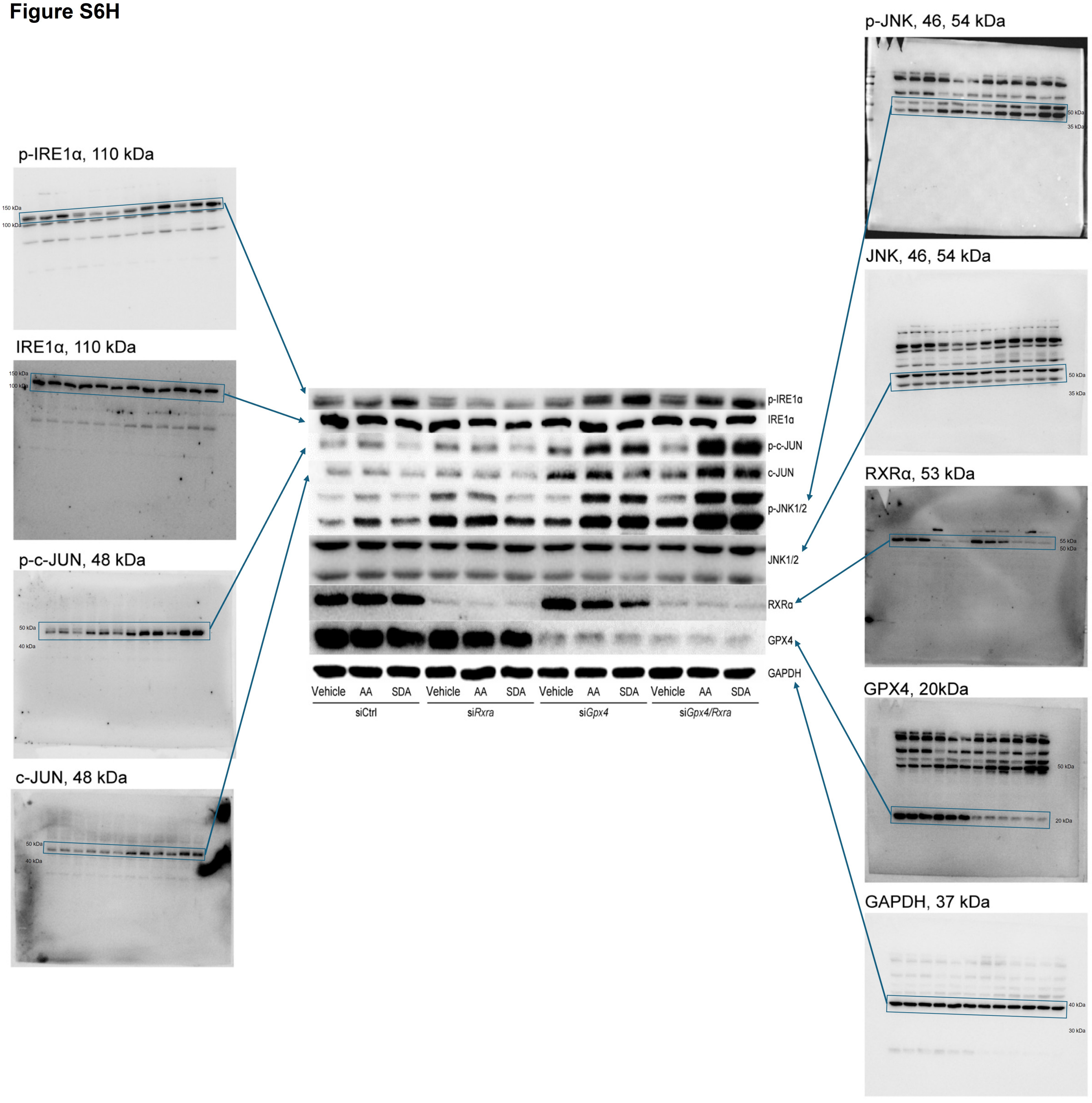

p-IRE1α, 110 kDa
150 kDa
100 kDa
IRE1α, 110 kDa
150 kDa
100 kDa
p-c-JUN, 48 kDa
50 kDa
40 kDa
c-JUN, 48 kDa
50 kDa
40 kDa
p-JNK, 46, 54 kDa
50 kDa
35 kDa
JNK, 46, 54 kDa
50 kDa
35 kDa
RXRα, 53 kDa
55 kDa
50 kDa
GPX4, 20kDa
50 kDa
20 kDa
GAPDH, 37 kDa
40 kDa
30 kDa
p-IRE1α
IRE1α
p-c-JUN
c-JUN
p-JNK1/2
JNK1/2
RXRα
GPX4
GAPDH
Vehicle
AA
SDA
Vehicle
AA
SDA
Vehicle
AA
SDA
Vehicle
AA
SDA
siCtrl
siRxra
siGpx4
siGpx4/Rxra
